# C/EBPβ expression decreases in cervical cancer and leads to tumorigenesis

**DOI:** 10.1186/s12885-023-10543-9

**Published:** 2023-01-24

**Authors:** Haichen Long, Yangyang Li, Huijuan Wang, Bingxuan Guo, Shuyan Song, Xiangyi Zhe, Hongtao Li, Dongmei Li, Renfu Shao, Zemin Pan

**Affiliations:** 1 Department of Biochemistry and Molecular Biology, School of Medicine, Xinjiang Endemic and Ethnic Disease and Education Ministry Key Laboratory, Shihezi University, Shihezi, 832002 Xinjiang China; 2 Department of Clinical Laboratory, First Affiliated Hospital of School of Medicine, Shihezi University, Shihezi, 832000 Xinjiang China; 3 Centre for Bioinnovation, School of Science, Technology and Engineering, University of the Sunshine Coast, Maroochydore, 4556 Australia

**Keywords:** C/EBPβ, Gene expression, Cervical cancer, Proliferation, Invasion, Migration

## Abstract

**Background:**

Cervical cancer is currently estimated to be the fourth most common cancer among women worldwide and the leading cause of cancer-related deaths in some of the world’s poorest countries. C/EBPβ has tumor suppressor effects because it is necessary for oncogene-induced senescence. However, C/EBPβ also has an oncogenic role. The specific role of C/EBPβ in cervical cancer as a tumor suppressor or oncoprotein is unclear.

**Objective:**

To explore the role of the C/EBPβ protein in cervical tumorigenesis and progression.

**Methods:**

Quantitative RT-PCR was used to analyze *C/EBPβ* (15 cervical cancer tissue samples and 15 corresponding normal cervical tissue samples)*, miR-661*, and *MTA1* mRNA expression in clinical samples (10 cervical cancer tissue samples and 10 corresponding normal cervical tissue samples). Immunohistochemistry was used to analyze C/EBPβ (381 clinical samples), Ki67 (80 clinical samples) and PCNA ( 60 clinical samples) protein expression. MALDI-TOF MassARRAY was used to analyze *C/EBPβ* gene methylation (13 cervical cancer tissues and 13 corresponding normal cervical tissues). Cell proliferation was analyzed by CCK-8 in cervical cancer cell lines. Western blotting and immunohistochemistry were performed to detect C/EBPβ protein expression levels, and mRNA expression was analyzed by quantitative RT-PCR analysis. Flow cytometry was performed to measure cell cycle distribution and cell apoptosis. Colony formation, Transwell, cell invasion, and wound healing assays were performed to detect cell migration and invasion.

**Results:**

C/EBPβ protein expression was significantly reduced in cervical cancer tissues compared with cervicitis tissues (*P* < 0.01). Ki67 protein and PCNA protein expression levels were significantly higher in cervical cancer tissues compared with cervicitis tissues. The rate of *C/EBPβ* gene promoter methylation of CpG12, 13, 14 and CpG19 in cervical cancer tissues was significantly increased compared with normal cervical tissue (*P* < 0.05). In addition, *C/EBPβ* was overexpressed in cervical cancer cells and this overexpression inhibited cell proliferation, migration, invasion, arrested cells in S phase, and promoted apoptosis.

**Conclusions:**

We have demonstrated that C/EBPβ decreased in cervical cancer tissues and overexpression of the *C/EBPβ* gene in cervical cancer cells could inhibit proliferation, invasion and migration.

**Supplementary Information:**

The online version contains supplementary material available at 10.1186/s12885-023-10543-9.

## Introduction

Cervical cancer is currently estimated to be the fourth most common cancer [1]among women worldwide and the leading cause of cancer-related deaths in some of the world’s poorest countries [2]. To date, organized and comprehensive cervical screening methods have been implemented mainly in high income countries, with the direct result that 85% of cervical cancer occurs in less developed regions [3]. According to the Agency for Research on Cancer database, the incidence and mortality of cervical cancer globally in 2012 was about 528,000 and 266,000, respectively [4, 5]. Originally named IL-6, C/EBPβ was first described in 1990 as a factor binding to the interleukin1 (IL-1) response element in IL-6 initiators and showed high C-terminal homology to C/EBPα [6]. The function of C/EBPβ in tumorigenesis is more complex than that of C/EBPα. Many of the biological properties of C/EBPβ are similar to those of C/EBPα, such as inhibiting proliferation and tumorigenesis and promoting differentiation. C/EBPβ inhibits cell proliferation by inhibiting the E2F target gene, which causes cell aging [7]. Expression of cancer proteins in primary cells often causes cell aging, which is a permanent state of cell growth prevention and is a tumor suppression mechanism. This cell suppression response, known as oncogene-induced senescence, is achieved by inducing p19Arf-p53 tumor suppression pathways and CDK inhibitors (such as p16Ink4a and p21CIP1) that activate the Rb-dependent checkpoint [8–10]. C/EBPβ has anti-cancer effects as it is necessary for oncogene-induced senescence [11]. C/EBPs are considered tumor inhibitors because they prevent cell growth, contribute to end-of-life differentiation of several cell types, and play a role in cell responses to DNA damage, nutritional deficiencies, hypoxia, and genotoxic factors. However, C/EBPs have the exact effect on cell proliferation and tumor development, and they are considered tumor suppressor proteins [12]. The role of C/EBPβ in cervical cancer is currently unclear. This research study aimed to understand the role of C/EBPβ in cervical cancer.

## Materials and methods

### Clinical sample collection

Clinical data and cervical squamous cancer specimens were collected from Kashgar People’s Hospital, Xinjiang Tumor Hospital, the First Affiliated Hospital and the Third Affiliated Hospital of the Medical College of Shihezi University, Xinjiang, China from January 2008 to May 2019. Cervical cancer samples were collected from patients with different pathological grades. None of the patients received radiotherapy or chemotherapy prior to cervical tissue collection. All histological diagnoses were confirmed by the hospital’s experienced pathologists. Each patient provided written informed consent. The study was approved by the Ethics Committee of Shihezi University of Medicine College. Samples obtained included cervical squamous cell carcinoma tissue, normal cervical tissue and para-carcinoma tissue. Fresh tissue samples were frozen immediately after removal and stored at -80 °C.

### Immunohistochemistry assay

Immunohistochemistry (IHC) staining was performed on 4 µm paraffin-embedded tissue specimens to detect protein expression levels of C/EBPβ, PCNA and Ki67. In short, formaldehyde fixed paraffin-embedded tissue and paired control tissue were sectioned at 4 µm. The sections were dewaxed for 25 min in xylene and rehydrated for 5 min in 100%, 95%, 80% and 70% ethanol. Antigen retrieval was carried out in 0.01 M citric acid buffer in the microwave for 3 min at high power followed by 10 min at low power. Slides were then incubated with 3% H_2_O_2_ at room temperature to block endogenous peroxidase activity. Slides were then washed in PBS solution and blocked with 10% goat serum (Zhong Shan Biotechnology, China) for 30 min. The slides were incubated overnight at 4 °C with rabbit anti-human C/EBPβ antibody (1:200; Millipore, USA), rabbit anti-human PCNA antibody (1:200; Millipore, USA), and rabbit anti-human Ki67 antibody (1:200; Millipore, USA). The slides were washed three times in PBS solution and incubated at room temperature with HRP-conjugated secondary antibodies for 30 min. All slides were dehydrated, chromogenically developed and counterstained with a solution of diaminobenzidine and 20% hematoxylin. IHC slides were reviewed and graded by two independent pathologists. Visual grading and stratification of staining intensity was performed on a scale of 0–3: Negative stain 0 (-), weak stain 1 ( +), moderate stain 2 (+ +), strong stain 3 (+ + +).

### The cDNA synthesis and quantitative RT-PCR

According to the manufacturer’s instructions, 100 mg of total RNA was extracted from tissues using the miRNeasy Mini Kit (Qiagen, Germany). RNA was quantified using a NanoDrop™ 2000 spectrophotometer (Thermo Fisher Scientific, Inc.). The standards for acceptable RNA were a A260/A280 ratio of 1.8–2.0. The cDNA was obtained using poly (A) polymerase reverse transcriptase primers (miRcute miRNA First-Strand cDNA Synthesis Kit, Tiangen Biotech, Inc., Beijing, China) at 37 °C 60 min, followed by 4 °C. Quantitative RT-PCR (qRT-PCR) was performed using the SYBR® Green PCR Kit (Qiagen, Germany) using a real-time PCR detection system (ABI 7500, Life Technology, USA). qRT-PCR amplification was performed with a denaturing step (95 °C 15 min), followed by 40 cycles of 94 °C for 15 s, 55 °C for 30 s, and 72 °C for 15 s. *GAPDH* was used as an internal control to normalize gene expression. The *GAPDH* gene was selected from the verification method described earlier as an appropriate reference gene and was used to determine the ΔCt value. Changes in gene expression were calculated using the 2^−ΔΔCt^ method. Gene expression results were analyzed based on the target gene/GAPDH ratio. The primers used were: *C/EBPβ*: F: 5′- CCTCGCAGGTCAAGAGCAAG -3′, R: 5′- GAACAAGTTCCGCAGGGTG -3′; *GAPDH*: F: 5′- TGTTGCCATCAATGACCCCTT -3′, R: 5′- CTCCACGACGTACTCAGCG -3′.

The qRT-PCR detection is performed at least three times.

### Western blotting

After cell lysis, the cell lysates were incubated in a water bath for 5 min at 100 °C for protein denaturation, and then centrifuged for 10 min at 12,000 × *g*. Separation of equal volume supernatant in 10% sodium dodecyl sulfate was performed with polyacrylamide gel electrophoresis. The proteins were transferred to polyvinylidene difluoride membranes (Beijing Solar Photoelectric Technology Co., Ltd., Beijing, China) using a semi-dry transfer. Membranes were blocked using 5% skim milk for 2 h, followed by primary antibody incubations overnight at 4 °C. Membranes were washed with Tris-buffered saline and Tween 20 three times at room temperature for 10 min each wash. Secondary antibody was incubated on the membranes for 2 h and the membranes were washed three times with Tris-buffered saline and Tween 20 for 10 min each wash. Blots were then washed with deionized water for 5 min. Blots were incubated with use ECL Plus, and imaging was performed using the ChemiDoc XRS + System (Bio-Rad, Hercules, CA, USA). Primary antibody dilution were as follows: anti-C/EBPβ (rabbit; 1:1,000; catalog # E299), anti-MTA1 (rabbit; 1:1,000; catalog # D40D1), and anti-β-Actin (rabbit; 1:1,000; catalog # 4967). Antibodies were purchased from Cell Signaling Technology, Inc. (USA). Western blot experiments were repeated five times.

### Cell culture and plasmid transfection

Human cervical cancer HeLa and SiHa cells were cultured in 10% fetal bovine serum (Sijiqing Technologies, Hangzhou, China), 100 U/mL penicillin and 100 mg/mL streptomycin (Solarbio, China) in Dulbecco’s Modified Eagle Medium (Gibco; Thermo Fisher Science, Inc., Waltham, VA, USA). Cells were kept at 37 °C in a humidified chamber containing 5% CO_2_. FuGENE HD (Roche Diagnostics) was used to transfect constructed plasmids into cell lines. The transfection mix was composed of 5 μl FuGENE HD, 2 μg plasmids, and 100 μl serum-free media. A 24-well plate was seeded with 1 × 10^5^ cells and cultured in complete media. After reaching 80% confluency, the cells were rinsed with PBS. Transfected cells were not cultured in penicillin or streptomycin. The experiment was repeated three times.

### Cell counting kit-8 assay

According to the manufacturer’s instructions, cells were inoculated into a 96-well plate at 5 × 10^3^ cells/well and cultured for 0, 12, 24, 36, 48 and 72 h after transfection. Cell proliferation was recorded at each timepoint using the Cell Counting Kit-8 (CCK-8) assay (Dojindo, Tokyo, Japan) for 72 h. The absorbance at 450 nm was detected using a microplate reader (Bio-Rad, USA), 2 h after culturing in the incubator (37 °C, 5% CO_2_). Set 4 repeated holes at each point in time and repeated the experiment at least 3 times.

### Colony formation assay

Cultured HeLa and SiHa cells were seeded into a 6-well plate at a concentration of 1 × 10^4^ cells per well and incubated at 37 °C for 2 weeks. The plates were then washed two times with PBS for 5 min each, followed by a methanol fixation for 15 min. Plates were then stained for 15 min in PBS with 0.1% Crystal Violet. Cell colonies were counted if they had at least 50 cells. The experiment was repeated three times.

### Cell cycle analysis

Cells were collected, washed with PBS, and fixed overnight in 70% ethanol at -20 °C. The ethanol-fixed cells were then pelleted, washed with ice-cold PBS, and then resuspended in 50 μg/mL propidium iodide (PI) and 100 μg/mL RNaseA. Cells were then cultured for 30 min at 37 °C. The cells were detected by flow cytometry. The percentage of cells in the G0-G1 phase, the S phase, and the G2 phase was analyzed. The experiment was repeated three times.

### Transwell migration and invasion assay

Cell migration and invasion assays were assessed using a transwell plate containing 8 µm perforated membrane (Corning Corporation, Corning, New York, USA). Transfected cells were resuspended at 5 × 10^4^ in serum-free medium and seeded into the upper chamber of the plate. Briefly, transfected cells were harvested, suspended in serum-free medium, and plated into the upper chamber for the migration or invasion assays, respectively. In addition, a culture base containing 20% fetal bovine serum was introduced into the lower chamber. Forty-eight hours after incubation at 37 °C, the cells in the upper chamber were removed. Cells on the lower surface were fixed and dyed, and the number of cells that had migrated or invaded in five random fields of view were measured using a microscope. The experiment was repeated three times.

### Wound healing assay

Five straight lines, 1 cm apart, were drawn on the back of a 6-well plate. Cell lines (2.5 × 10^5^ cells/ml in 2 mL) were added to the culture dish and cultured for 24 h. Scratches were made in the cell layer using 200 μL pipette tips perpendicular to five of the pre-drawn lines. The cells were washed with PBS and 2 mL of serum-free culture media were added to the wells. The migration of the cells at specified times (0, 24, and 48 h) was observed using an inverted microscope. The experiment was repeated three times.

### Annexin V-PI double staining assay

Cells were transferred to a 1.5 mL centrifuge tube after transfection for 48 h. Cells were resuspended in 0.5 mL of binding buffer. Subsequently, 5 μL of PI and 5 μL of Annexin V-EGFP were added to the centrifuge tube. The cells were incubated for 15 min at 25 °C and then measured with a flow cytometer.

### MassARRAY assay

DNA was extracted from tissue samples using the QIAamp DNA Mini Kit (Qiagen, Germany). Bisulfite was added to the EZ DNA Methylation Kit (Sequenom, USA). The PCR reaction was set up with a total volume of 5 μl and the following: 5 ng template, 0.5 U Hot Star Taq polymerase (Qiagen), forward and reverse primers, (0.5 μL, 10 pmol), 0.5 μL of 10 × PCR buffer, 0.4 μL of MgCl_2_, 0.5 μL of ddH_2_O, and 0.1 μL of 25 mM dNTP. PCR amplification consists of a denaturation step (95 °C for 15 min), followed by 45 cycles of 94 °C for 20 s, 62 °C for 30 s, 72 °C for 60 s, then 72 °C for 3 min. Shrimp alkaline phosphatase (2 μL) was then added to each reaction. The samples were centrifuged at 3,000 rpm for 5 min, cultured at 37 °C for 20 min, then 85 °C for 5 min. For the RNA transcription volume of 5 μL, the following were included: 0.89 μL of 5 × T cleavage & Polymerase Buffer, 3.15 μL of RNase-free ddH_2_O, 0.24 μL of T cleavage mix, 0.44 μL of T7 RNA & DNA polymerase, 0.06 μL of RNAase A, and 0.22 μL of 100 mM DTT. After incubating the samples for 3 h at 37 °C, 6 mg Clean Resin (Sequenom) was added to desalt the RNA.

### Statistics assay

Statistical analysis was performed with SPSS 17.0 software. *P* < 0.05 signified statistical significance. Protein levels were compared between cervicitis tissues and cervical cancer tissues using non-parametric tests. MALDI-TOF MassARRAY data were analyzed using a Wilcoxon Rank Sum Test. The remaining data was analyzed using a Student’s *t*-test.

## Results

### C/EBPβ, Ki67 and PCNA protein level in cervical carcinoma tissues and chronic cervicitis tissues

**Fig. 1:**
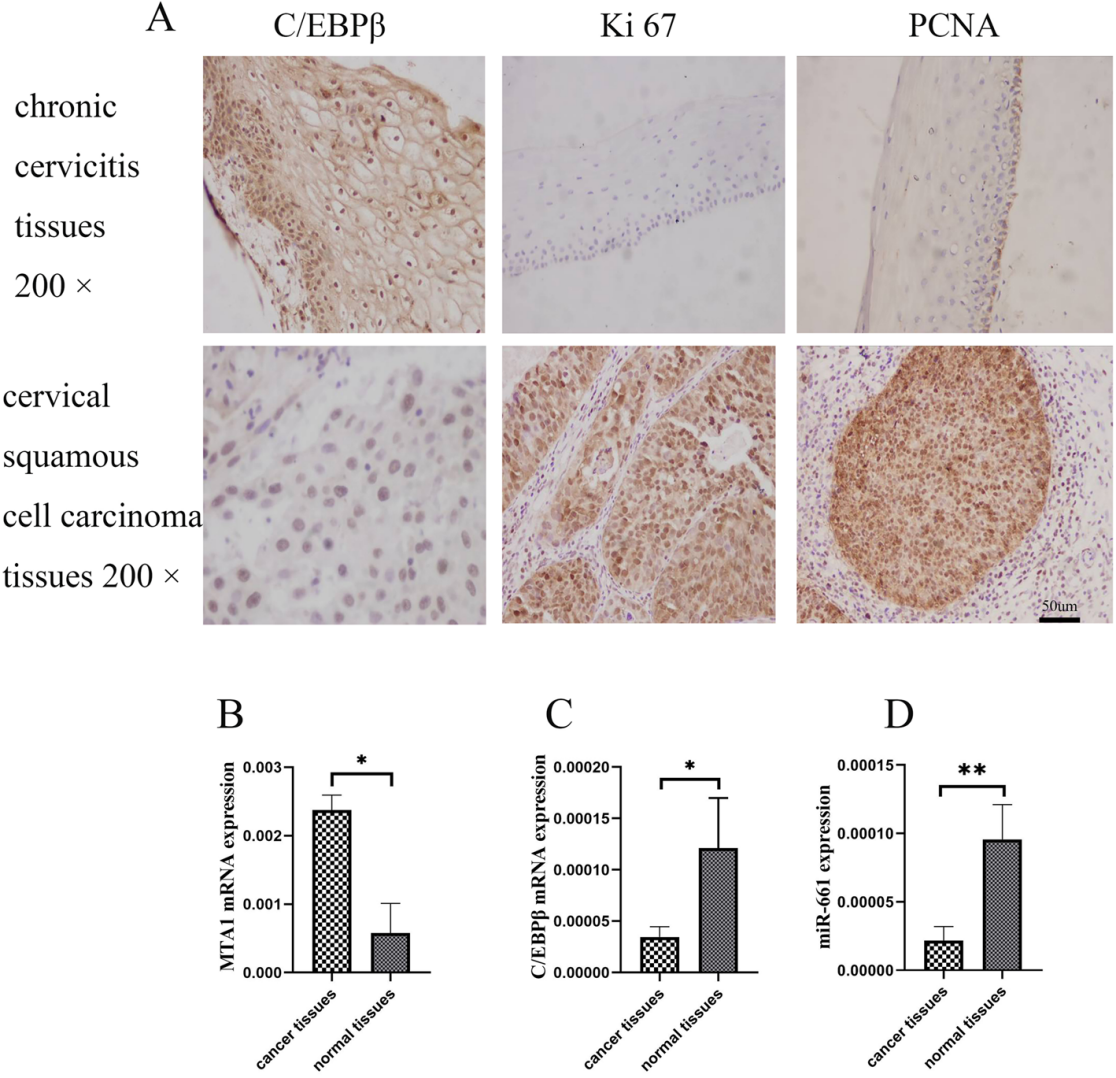
Immunohistochemistry (IHC) and qRT-PCR in clinical cervical samples. **A** IHC (× 200 magnification) of C/EBPβ, Ki67, and PCNA protein in chronic cervicitis tissue and cervical cancer tissue. **B**, **C**, **D** qRT-PCR of *MTA1* mRNA, *C/EBPβ* mRNA and *miR-661 *in cervical cancer tissues and normal cervical tissues. The Student’s *t*-test test was used to compare the two groups of single variable data, single factor analysis of variance comparing multiple groups of data, nonparametric test to compare the variance not neat, count data using chi-square test. Ns: Not significant. ^*^P < 0.05, ^**^P < 0.01. The experiment was repeated three times. **B** Expression of *MTA1* was significantly higher in 10 cervical cancer tissues compared with 10 normal cervical tissues (expression ratio: *MTA1*/*GAPDH*). **C** *C/EBPβ* was significantly downregulated in 15 cervical cancer tissues compared with 15 normal cervical tissues (expression ratio: *C/EBPβ*/*GAPDH*). **D** *miR-661* was significantly downregulated in 10 cervical cancer tissues compared with 10 normal cervical tissues (expression ratio: *C/EBPβ*/*GAPDH*)

We conducted IHC on 381 clinical samples (Fig. 1A, Tables 1, 2 and 3).

**Table 1:** The expression of C/EBPβ protein in tissues with chronic cervicitis and cervical cancer

Tissues	Number	-	+	+ +	+ + +
Chronic cervicitis tissues	172	19	30	70	53
Cervical squamous cell carcinoma tissues	209	47	57	56	49

Data were analyzed using nonparametric test,chi-square test

χ^2 ^= 18.552 *P* < 0.01

**Table 2:** The expression of Ki67 protein in tissues with chronic cervicitis and cervical cancer

Tissues	Number	-	+	+ +	+ + +
Chronic cervicitis tissues	32	17	6	2	7
Cervical squamous cell carcinoma tissues	48	17	3	3	25

Data were analyzed using nonparametric test,chi-square test

χ^2^ = 8.464 *P* < 0.05

**Table 3:** The expression of PCNA protein in tissues with chronic cervicitis and cervical cancer

Tissues	Number	-	+	+ +	+ + +
Chronic cervicitis tissues	27	11	4	11	1
Cervical squamous cell carcinoma tissues	38	1	3	19	15

Data were analyzed using nonparametric test,chi-square test

χ^2^ = 22.367 *P* < 0.01

Samples had negative stain (-), samples had weak stain ( +), samples had moderate stain (+ +), samples had strong stain (+ + +).

C/EBPβ protein was measured in 172 cervicitis tissues and 209 cervical cancer tissues. For cervicitis tissues, 19 samples (11%) (-), 30 samples (17.5%) (+), 70 samples (40.7%) (++), and 53 (30.8%) (+++). For cervical cancer tissues, 47 samples (22.5%) (-), 57 samples (27.3%) (+), 56 samples (26.8 %) (++), and 49 samples (23.4%) (+++). C/EBPβ protein showed low expression in cervical cancer tissues and high expression in cervicitis tissues [χ^2^ = 18.552, *P* < 0.01 (Table 1)].

Ki67 protein was measured in 32 cervicitis tissues and 48 cervical cancer tissues by IHC. In the cervicitis tissues, 17 samples (53.1%) (-), 6 samples (18.8%) ( +), 2 samples (6.2%) (+ +), and 7 samples (21.9%) (+ + +). In the cervical cancer tissues, 17 samples (35.4%) (-), 3 samples (6.25%) ( +), 3 samples (6.25%) (+ +), and 25 samples (52.1%) (+ + +). Ki67 protein was expressed more in cervical cancer tissues than in cervicitis tissues [χ^2^ = 8.464, *P* < 0.05 (Table 2)].

PCNA protein was also measured in 27 cervicitis tissues and 38 cervical cancer tissues by IHC. For cervicitis tissues, 11 samples (40.7%) (-), 4 samples (14.9%) ( +), 11 samples (40.7%) (+ +), and 1 sample (3.7%) (+ + +). For cervical cancer tissues, 1 sample (2.6%) (-), 3 samples (7.9%) ( +), 19 samples (50%) (+ +), and 15 samples (39.5%) (+ + +). Proliferating cell nuclear antigen (PCNA) protein was highly expressed in cervical cancer tissues compared with cervicitis tissues [χ^2^ = 22.367, *P* < 0.01 (Table 3)]. C/EBPβ was downregulated in cervical cancer tissues compared with cervicitis tissues. Ki67 and PCNA were upregulated in cervical cancer tissues compared with cervicitis tissues and correlated with cancer cell proliferation. Therefore, C/EBPβ could potentially modulate cervical cell proliferation.

### C/EBPβ, MTA1 and miR-661 expression level in cervical carcinoma tissues and chronic cervicitis tissues

The qRT-PCR was performed on clinical samples (Fig. 1B-D). MTA1 and miR-661 expression were measured in 10 cervical cancer tissue samples and 10 corresponding normal cervical tissue samples. miR-661 was downregulated in cervical cancer tissues compared with normal cervical tissues. On the contrary, MTA1 was significantly upregulated in cervical cancer compared with normal cervical tissues (*P* < 0.05). C/EBPβ mRNA was tested in 15 cervical cancer samples and 15 corresponding normal cervical tissue samples. C/EBPβ was significantly downregulated in cervical cancer tissues compared with normal cervical tissues (*P* < 0.05). The result showed that C/EBPβ and miR-661 were expressed in cervical cancer tissues significantly lower than in normal cervical tissues. However, MTA1, a metastasis related protein, was significantly overexpressed in cervical cancer tissues compared with normal cervical tissues.

### Methylation in the promoter of *C/EBPβ* gene analysis

**Fig. 2:**
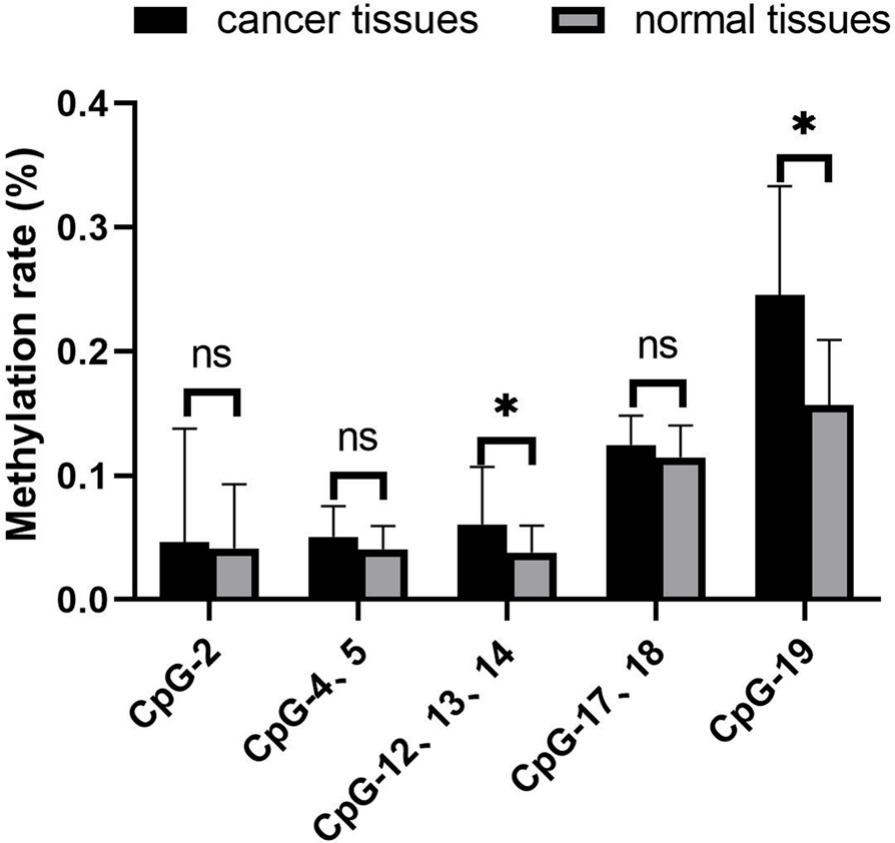
The methylation rate of the *C/EBPβ* gene promoter in cervical cancer tissues and normal cervical tissues. *C/EBPβ* promoter methylation was measured in 13 cervical cancer tissues and 13 corresponding normal cervical tissues. Data were analyzed using a Wilcoxon Rank Sum Test. Ns: not significant. ^*^P < 0.05, ^**^P < 0.01. The experiment was repeated three times

*C/EBPβ* promoter methylation was measured in 13 cervical cancer tissues and 13 corresponding normal cervical tissues (Fig. 2). The rate of methylation of CpG-12, 13, 14 and CpG-19 was significantly higher in cervical cancer tissues than in normal cervical tissues (*P* < 0.05). Promoter of the *C/EBPβ* gene in cervical cancer tissues has hypermethylation sites.

### *C/EBPβ* gene construct transfected into HeLa and SiHa cells, C/EBPβ, MTA1 and miR-661 expression level in HeLa and SiHa cells

**Fig. 3:**
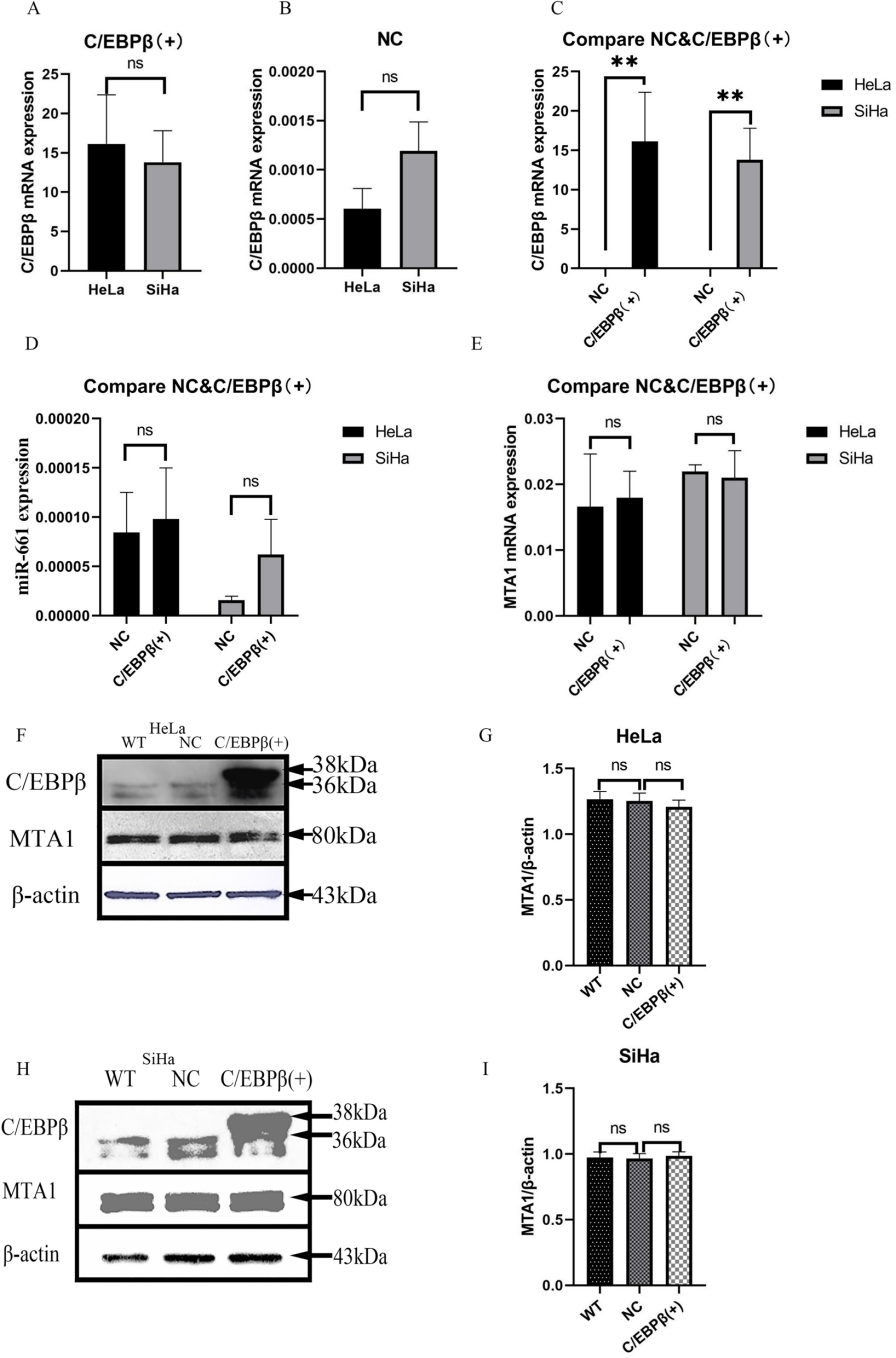
The qRT-PCR and western blotting of cervical cancer cell lines. C/EBPβ ( +): pcDNA3.1-C/EBPβ plasmid transfection; WT: without any transfection; NC: negative control pcDNA3.1 plasmid transfection. The Student’s *t*-test test was used to compare the two groups of single variable data, single factor analysis of variance comparing multiple groups of data, nonparametric test to compare the variance not neat, count data using chi-square test. Ns: not significant. ^*^P < 0.05, ^**^P < 0.01. **A**, **B**, **C**, **D**, **E** qRT-PCR of the expression of *C/EBPβ* mRNA in Hela and SiHa cells (expression ratio: *C/EBPβ*/*GAPDH*). The experiment was repeated three times. **A** For measuring *C/EBPβ* mRNA expression in overexpression cells, pcDNA 3.1-C/EBPβ plasmid was transfected into HeLa and SiHa cells for 48 h. **B** For measuring *C/EBPβ* mRNA expression in control cells, pcDNA3.1-Mock plasmid was transfected into HeLa and SiHa cells for 48 h. **C** Merged (A,B) mRNA expression data in pcDNA 3.1-C/EBPβ and pcDNA3.1-Mock-transfected cells. **D** For measuring *miR-661 *expression in overexpression cells, plasmids were transfected into HeLa cells and SiHa cells for 48 h. **E **For measuring *MTA1* mRNA expression in overexpression cells, plasmids were transfected into HeLa and SiHa cells for 48 h. **F**, **G**, **H**, **I** Western blotting to detect effects of C/EBPβ overexpression on MTA1 expression. The experiment was repeated five times. **F**, **G** Plasmid transfection in HeLa cells after 72 h. **H**, **I** Plasmid transfection in SiHa cells after 72 h

The qRT-PCR was performed on HeLa and SiHa cells after *C/EBPβ* gene transfection (Fig. 3A-E). *C/EBPβ* mRNA in overexpression cells was significantly higher compared to the control groups (*P* < 0.01). *miR-661* expression and *MTA1* mRNA levels, however, did not show a significance change in HeLa and SiHa cells after *C/EBPβ* gene overexpression. The qRT-PCR results showed that the C/EBPβ plasmids were successful in overexpression in cervical cancer cells.

### *C/EBPβ* gene construct transfected into HeLa and SiHa cells, C/EBPβ, MTA1 protein level in HeLa and SiHa cells

Western blot was conducted on HeLa and SiHa cell lysates post-transfection (Fig. 3F-I). C/EBPβ protein was upregulated compared to the control groups after *C/EBPβ* gene transfection. However, MTA1 protein was not significantly different. These western blot results demonstrated that the *C/EBPβ* gene was successfully transfected into these cells. C/EBPβ protein overexpression did not influence MTA1 protein levels. When reading the literature [13], we found that miR-661 can be regulated by C/EBPα, and C/EBPβ and C/EBPα are the same family. We wanted to verify whether miR-661 can also be regulated by C/EBPβ. The results showed that the C/EBPβ does not through the miR-661-MTA1 pathway.

### *C/EBPβ* gene construct transfected into HeLa and SiHa cells, inhibit cell growth, decrease cell migration, and promote cell apoptosis

**Fig. 4:**
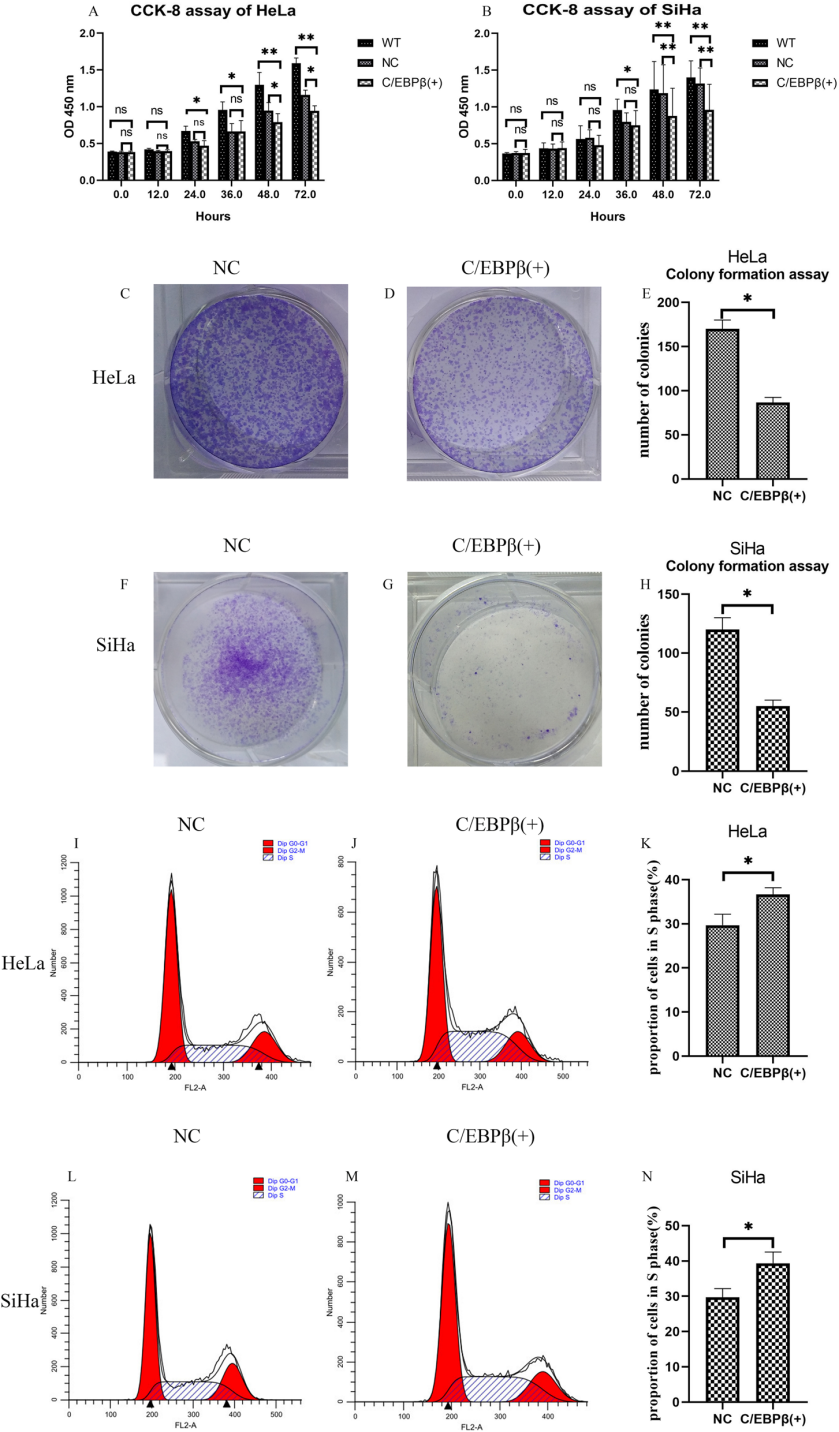
Cell proliferation and cell cycle assay. C/EBPβ ( +): pcDNA3.1-C/EBPβ plasmid transfection; WT: without any transfection; NC: negative control pcDNA3.1 plasmid transfection. The Student’s *t*-test test was used to compare the two groups of single variable data, single factor analysis of variance comparing multiple groups of data, nonparametric test to compare the variance not neat, count data using chi-square test. Ns: not significant. ^*^P < 0.05, ^**^P < 0.01. All experiments were repeated three times. **A**, **B** Effects of C/EBPβ overexpression on cell proliferation in HeLa and SiHa cells. **A**) HeLa cell proliferation patterns were measured using the CCK-8 assay at specified timepoints (0, 12, 24, 36, 48 and 72 h) after transfection. **B** SiHa cell proliferation patterns were detected using the CCK-8 assay at specified timepoints (0, 12, 24, 36, 48 and 72 h) after transfection. **C**, **D**, **E**, **F**, **G**, **H** Effect of C/EBPβ overexpression on HeLa and SiHa cell colony formation. **C**, **D**, **E** HeLa colony formation assay. **F**, **G**, **H** SiHa colony formation assay. **I**, **J**, **K**, **L**, **M**, **N** Effects of C/EBPβ overexpression on the cell cycle in HeLa and SiHa cells. **I**, **J**, **K** Cell cycle assay in HeLa cells. **L**, **M**, **N** Cell cycle assay in SiHa cells

The CCK-8 assay was conducted on HeLa and SiHa cells after transfection with plasmids (Fig. 4A-B). Absorbance at 450 nm was measured after transfection at 0, 12, 24, 36, 48 and 72 h post-assay set-up. Cell proliferation was significantly decreased in *C/EBPβ-*transfected cervical cancer cells compared to the control groups (*P* < 0.01). The CCK-8 data illustrated that C/EBPβ overexpression in cervical cancer cells inhibited proliferation.

The colony formation assay was performed with HeLa and SiHa cells post-transfection (Fig. 4C-H). The number of colonies were significantly reduced compared to the control groups after transfection with the *C/EBPβ* gene (*P* < 0.05). These data showed that C/EBPβ overexpression in cervical cancer cells inhibited proliferation.

Cell cycle analysis was performed on HeLa and SiHa cells after transfection with *C/EBPβ* gene plasmids (Fig. 4I-N). The proportion of cells in S phase was significantly increased compared to the control groups after transfection with the *C/EBPβ* gene (*P* < 0.05). The results illustrated that C/EBPβ overexpression in cervical cancer cells arrested cells in S phase.

**Fig. 5:**
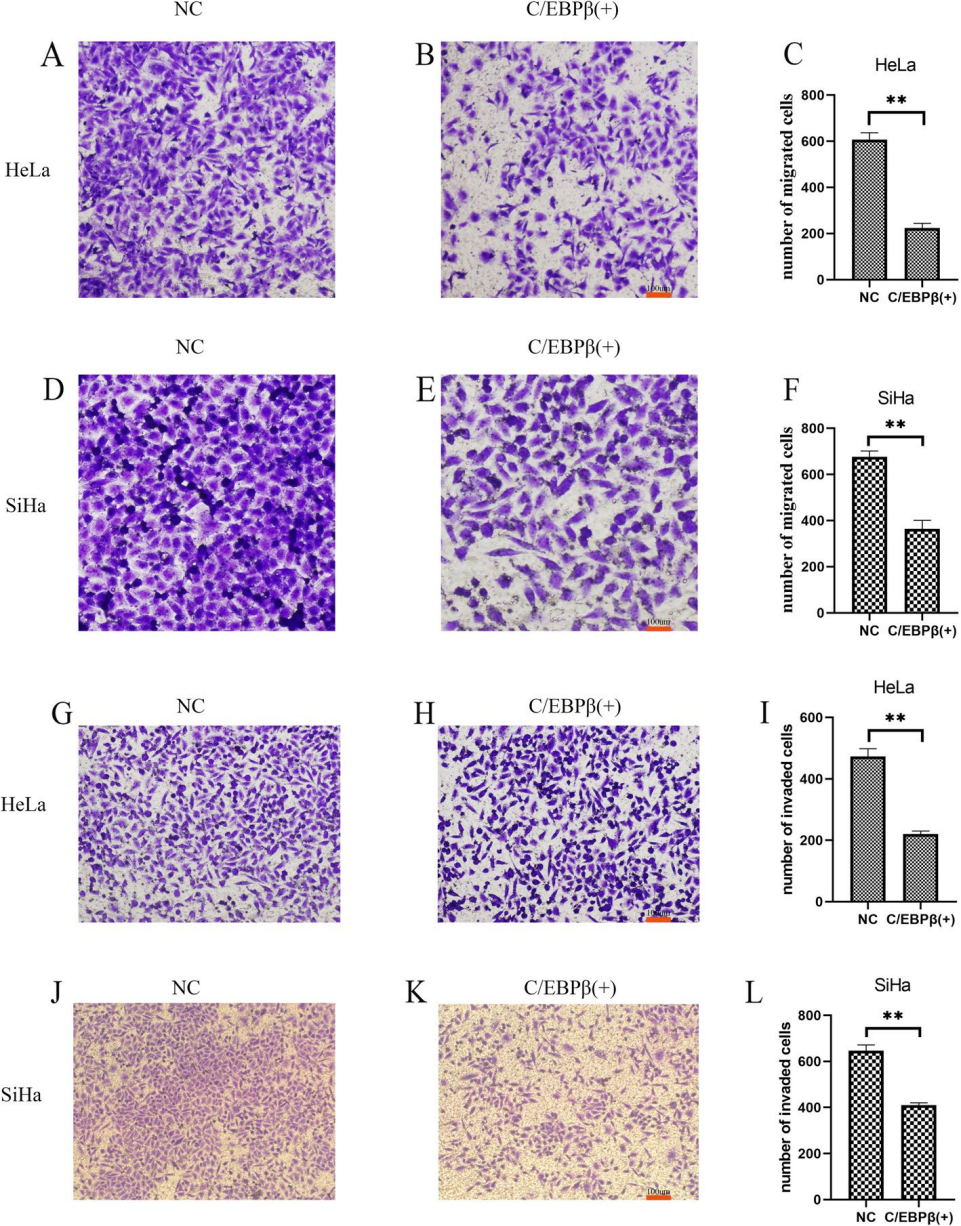
Cell migration and invasion assays. C/EBPβ ( +): pcDNA3.1-C/EBPβ plasmid transfection; NC: negative control pcDNA3.1 plasmid transfection. The Student’s *t*-test test was used to compare the two groups of single variable data, single factor analysis of variance comparing multiple groups of data, nonparametric test to compare the variance not neat, count data using chi-square test. Ns: Not significant. ^*^P < 0.05, ^**^P < 0.01. All experiments were repeated three times. **A**, **B**, **C**, **D**, **E**, **F** Effects of C/EBPβ overexpression on HeLa and SiHa cell migration. **A**, **B**, **C** HeLa cell migration assay. **D**, **E**, **F** SiHa cell migration assay. **G**, **H**, **I**, **J**, **K**, **L** Overexpression of C/EBPβ inhibits the invasive ability of HeLa and SiHa cells. **G**, **H**, **I** HeLa cell invasion assay. **J**, **K**, **L** SiHa cell invasion assay

The transwell assay was conducted with HeLa and SiHa cells after transfection with *C/EBPβ* gene plasmids (Fig. 5A-F). The number of migrated cells was significantly less with *C/EBPβ* overexpression compared with the control group (*P* < 0.01). The results showed that *C/EBPβ* gene overexpression inhibited migration in cervical cancer cells.

A cell invasion assay was conducted with HeLa and SiHa cells after transfection with *C/EBPβ* gene plasmids (Fig. 5G-L). The number of invaded cells was significantly less with *C/EBPβ* overexpression compared to the control groups (*P* < 0.01). Our results demonstrated that *C/EBPβ* gene overexpression inhibited the invasion in cervical cancer cells.

**Fig. 6:**
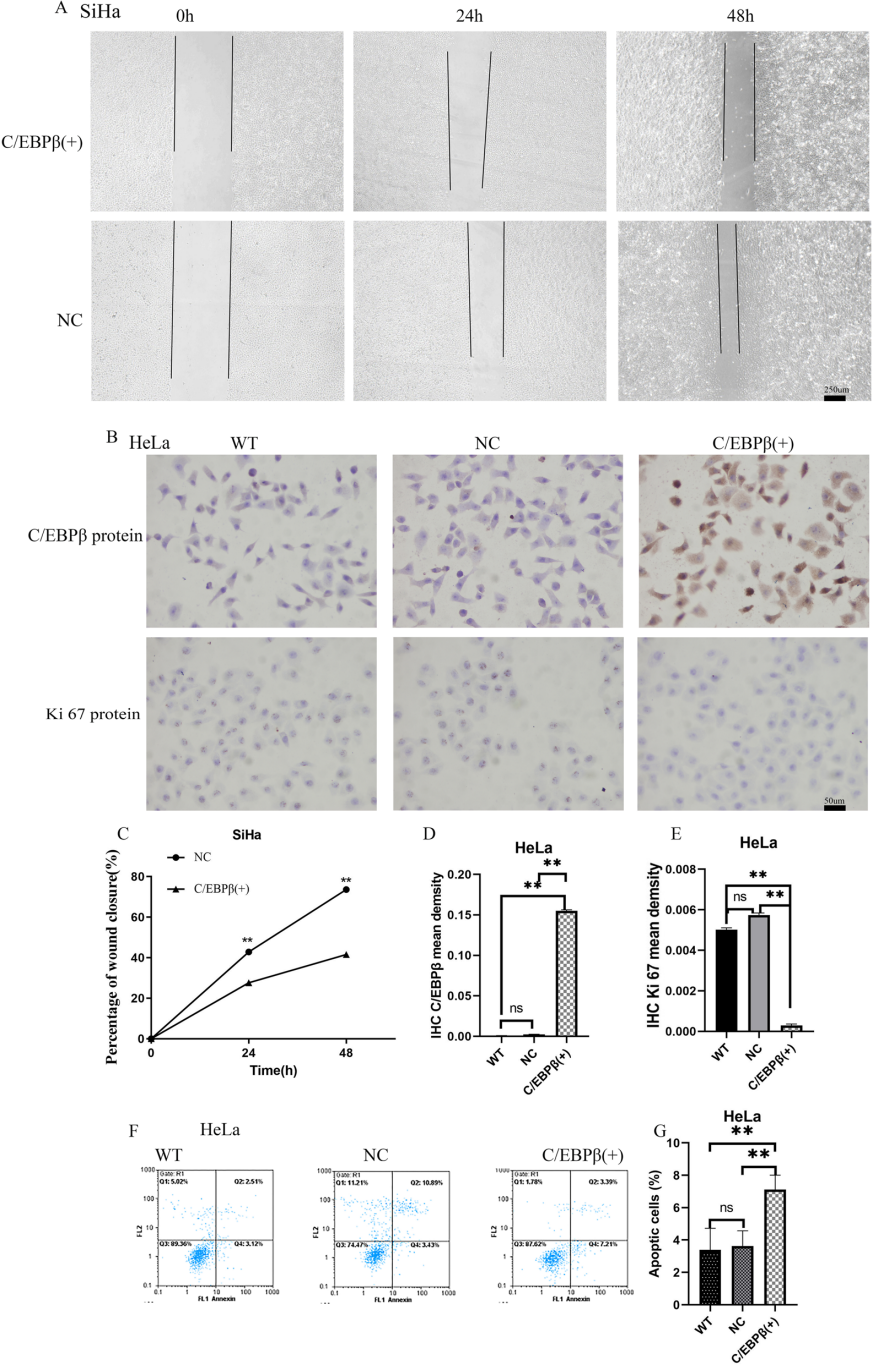
Wound healing assay, cell immunohistochemistry (IHC), and cell apoptotic assay. C/EBPβ ( +): pcDNA3.1-C/EBPβ plasmid transfection; WT: without any transfection; NC: negative control pcDNA3.1 plasmid transfection. The Student’s *t*-test test was used to compare the two groups of single variable data, single factor analysis of variance comparing multiple groups of data, nonparametric test to compare the variance not neat, count data using chi-square test. Ns: Not significant. ^*^P < 0.05, ^**^P < 0.01. All experiments were repeated three times. **A**, **C** Wound healing assay measured the effects of C/EBPβ overexpression on SiHa cell migration. Wound healing assay of SiHa cells after plasmid transfection at specified timepoints (0, 24, 48 h). **B**, **D**, **E** HeLa cell IHC (× 200 magnification) to measure the effects of C/EBPβ overexpression on Ki67 expression. **F**, **G** Annexin V-PI double staining for measurement of apoptosis in each treatment group. Effect of C/EBPβ overexpression on HeLa cell apoptosis. Annexin V-PI double staining of plasmid transfection of HeLa cells. Percentage of apoptotic cells in each group

A wound healing assay was performed with SiHa cells after transfection with *C/EBPβ* gene plasmids (Fig. 6A, C). The scratches were significantly wider at 24 h and 48 h with *C/EBPβ* overexpression compared to the control groups (*P* < 0.01). The results showed that *C/EBPβ* gene overexpression inhibited migration in cervical cancer cells.

IHC was performed on HeLa cells after transfection with *C/EBPβ* gene plasmids (Fig. 6B, D-E). C/EBPβ protein levels were higher with C/EBPβ overexpression compared to the control groups. However, Ki67 protein levels decreased in the *C/EBPβ* gene transfection group. These data demonstrated that C/EBPβ protein overexpression reduced Ki67 protein expression and decreased HeLa cell proliferation.

Annexin V-PI double staining was performed on HeLa cells after plasmid transfection (Fig. 6F-G). The apoptosis rate was significantly higher after transfection with the *C/EBPβ* gene compared to the control groups (*P* < 0.01). The results showed that C/EBPβ overexpression promoted apoptosis of cervical cancer cells.

## Discussion

C/EBPβ plays an important role in the cellular differentiation and regulation of stress and metabolism [14]. Increased expression of the C/EBPβ protein in myeloid progenitor cells increases the expression of miR-21 and miR-181b [15]. In myeloid cells, C/EBPβ deficiency inhibits myeloid-derived suppressor cell development [16]. Experiments have shown that inhibiting the activity of C/EBPβ in mice could impede the differentiation of adipocytes [17]. AMPK signaling may indirectly suppress C/EBPβ factors and C/EBPβ may regulate cell differentiation [18]. C/EBPβ plays an important role in osteoclast cell differentiation [19]. C/EBPβ has tumor inhibition and anti-proliferation activity in some tumors, but overexpressed C/EBPβ increases tumor invasion in some cancers [20]. C/EBPβ overexpression has been observed in aging mice [21]. C/EBPβ may be associated with aging [22]. C/EBPβ plays a role in suppressing cancer in a variety of cancers [23]. We conducted IHC on 381 clinical samples (Fig. 1A, Tables 1, 2 and 3) and found a significant difference between the low expression of C/EBPβ protein in cervical cancer tissues and the high expression in cervicitis tissues (χ^2^ = 18.552, *P* < 0.01). Ki67 and PCNA proteins associated with proliferation were highly expressed in cervical cancer tissues and were decreased in cervicitis tissues, and this difference was significant (Ki67: χ^2^ = 8.464, *P* < 0.05; PCNA: χ^2^ = 22.367, *P* < 0.01).

Transfection with *C/EBPβ* gene plasmids into cells was demonstrated to be successful through qRT-PCR (Fig. 3A-E), western blotting (Fig. 3F-I) and cell IHC (Fig. 6B, D-E). C/EBPβ plasmid-transfected cells overexpressed *C/EBPβ* mRNA and C/EBPβ protein. We found that the expression of the proliferation-related Ki67 protein decreased compared to the control group in cells after overexpression of the *C/EBPβ* gene (Fig. 6B, E). Overexpression of C/EBPβ in cervical cell lines significantly decreased cell proliferation (Fig. 4A-B, *P* < 0.01), significantly reduced the number of colonies (Fig. 4C-H, *P* < 0.05), and also significantly arrested cells in S phase (Fig. 4I-N, *P* < 0.05). Overexpression of C/EBPβ significantly decreased the number of migrated cells (Fig. 5A-F, *P* < 0.01), significantly reduced the number of invaded cells (Fig. 5G-L, *P* < 0.01), and resulted in significantly wider scratches (Fig. 6A, C, *P* < 0.01). Through these experiments, it was demonstrated that C/EBPβ overexpression in cervical cancer cells inhibited the cell proliferation, migration, and invasion of cervical cancer cells, promoted apoptosis, and arrested these cells in S phase.

The qRT-PCR analysis of clinical samples (Fig. 1B-C) showed that *C/EBPβ* mRNA was significantly downregulated in cervical cancer tissues compared with normal cervical tissues (*P* < 0.05). *MTA1* gene expression was lower in normal cervical tissues than in cervical cancer tissues (*P* < 0.05). It was reported that C/EBPα could play the role of tumor suppressor through the miR-661-MTA1 pathway [13]. C/EBPβ and C/EBPα belong to the C/EBP family. We therefore wanted to test whether C/EBPβ also played the role of tumor suppressor through the miR-661-MTA1 pathway. However, our experiments showed that C/EBPβ played the role of tumor suppressor in cervical cancer, but not through the miR-661-MTA1 pathway. There was not a significant increase in MTA1 expression in C/EBPβ-overexpressing cervical cancer cells (Fig. 3). In the C/EBPβ methylation assessment of 26 clinical samples (13 cervical cancer tissues and 13 corresponding normal cervical tissues), it found that the rate of methylation of CpG12, 13, 14 and CpG19 in cervical cancer tissues was significantly higher than in normal cervical tissue (Fig. 2, *P* < 0.05). It was possible that methylation at these positions reduced the expression of *C/EBPβ* in cervical cancer tissues.

## Conclusions

Our experiments have shown that C/EBPβ overexpression in cervical cancer cells inhibited the cell proliferation, migration, and invasion of cervical cancer cells, promoted apoptosis, and arrested these cells in S phase. Promoter of the *C/EBPβ* gene in cervical cancer tissues has hypermethylation sites. C/EBPβ did not appear to signal through the MTA1 pathway in cervical cancer. The exact pathway of C/EBPβ as a tumor suppressor in cervical cancer is not clear, however. C/EBPβ may play the role of tumor suppressor in cervical cancer.

## Supplementary Information

**Additional file 1:**

## Data Availability

All data generated or analysed during this study are included in this published article [and its supplementary information files]. If someone wants to request the data from this study please contacted Haichen Long, e-mail: 2216177915@qq.com.
