# Supplementary figures and images for: C/EBPβ expression decreases in cervical cancer and leads to tumorigenesis
Source: BMC Cancer. 2023 Jan 24;23:79. doi: 10.1186/s12885-023-10543-9 (PMC9872280; doi:10.1186/s12885-023-10543-9)

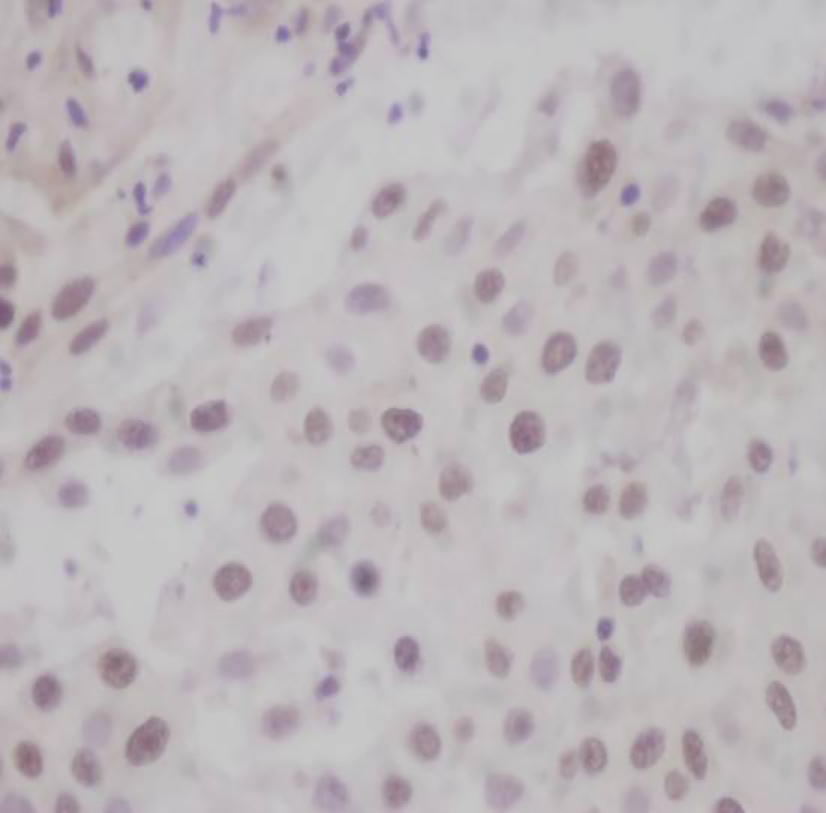

Supplement: Supplementary file 1 — Additional file 1: [file 12885_2023_10543_MOESM1_ESM.zip › Fig1 CEBPB protein in cervical cancer tissue 300ppi original.jpg]

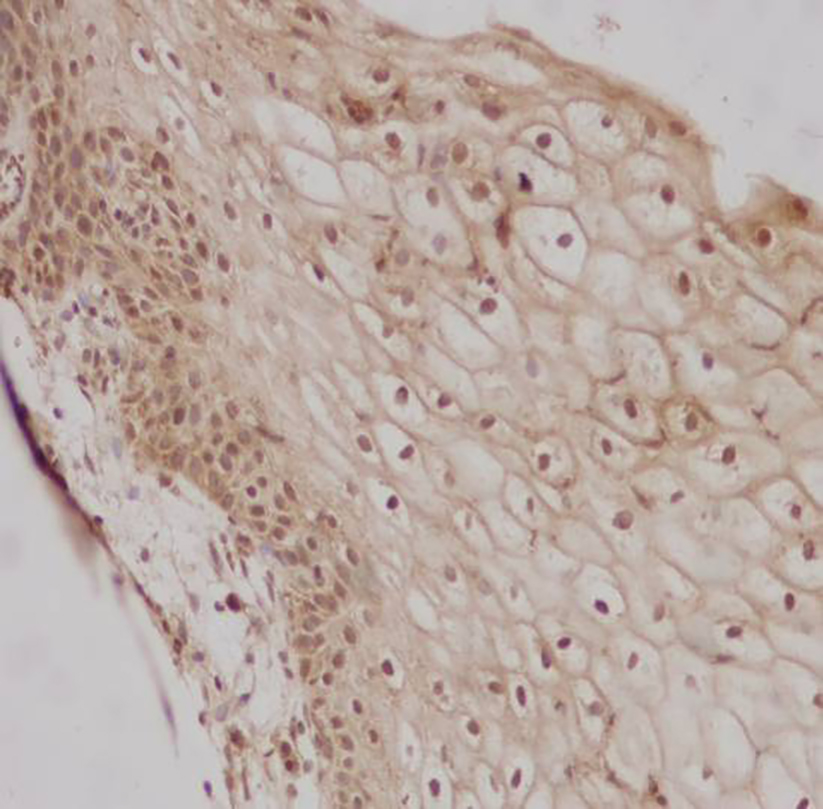

Supplement: Supplementary file 1 — Additional file 1: [file 12885_2023_10543_MOESM1_ESM.zip › Fig1 CEBPB protein in chronic cervicitis tissue 300ppi original.jpg]

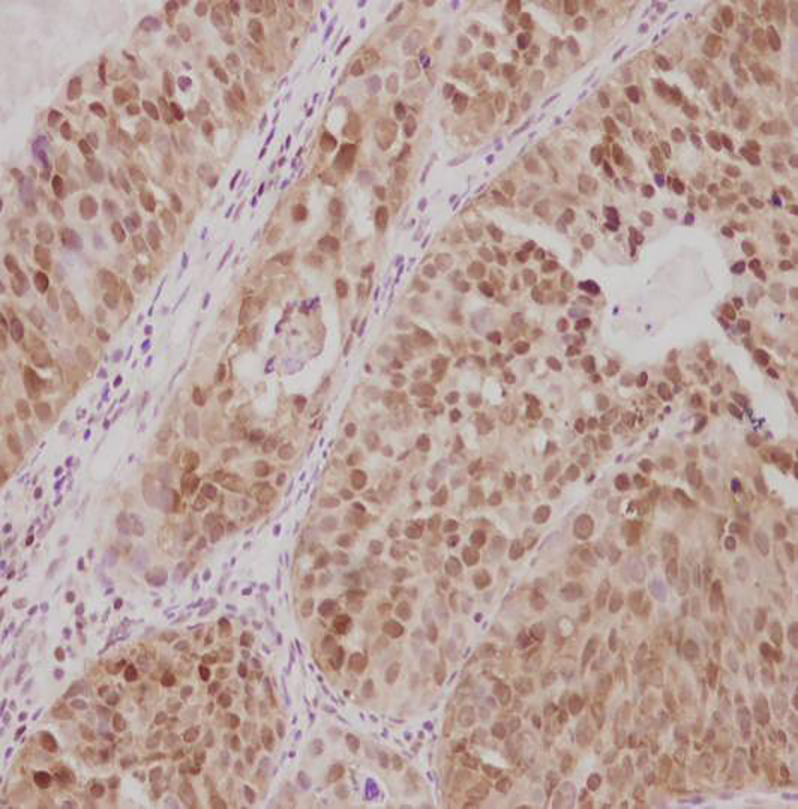

Supplement: Supplementary file 1 — Additional file 1: [file 12885_2023_10543_MOESM1_ESM.zip › Fig1 Ki67 protein in cervical cancer tissue 300ppi original.jpg]

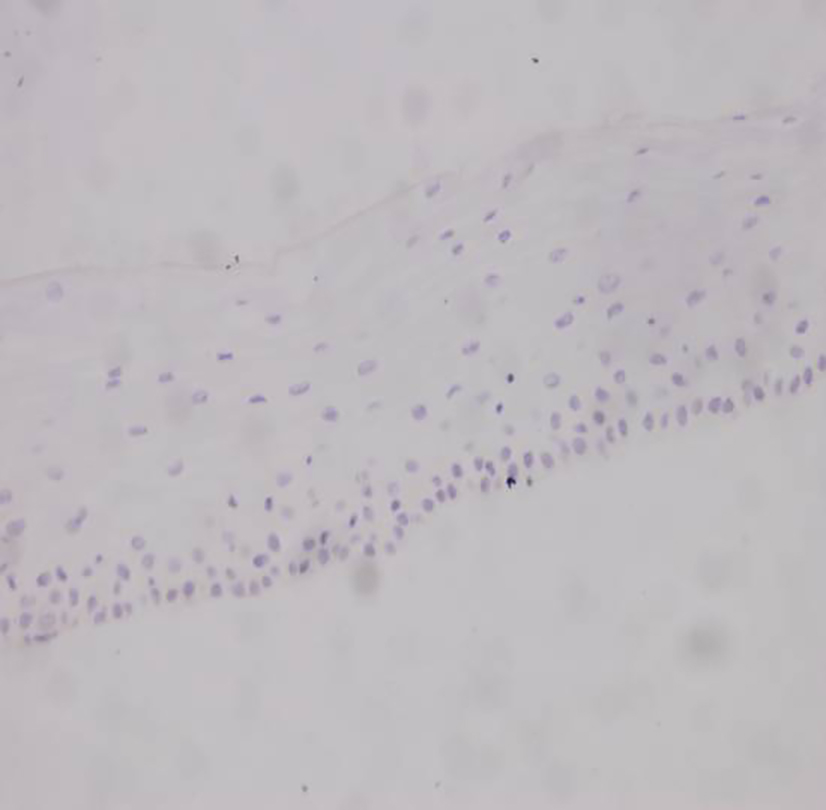

Supplement: Supplementary file 1 — Additional file 1: [file 12885_2023_10543_MOESM1_ESM.zip › Fig1 Ki67 protein in chronic cervicitis tissue 300ppi original.jpg]

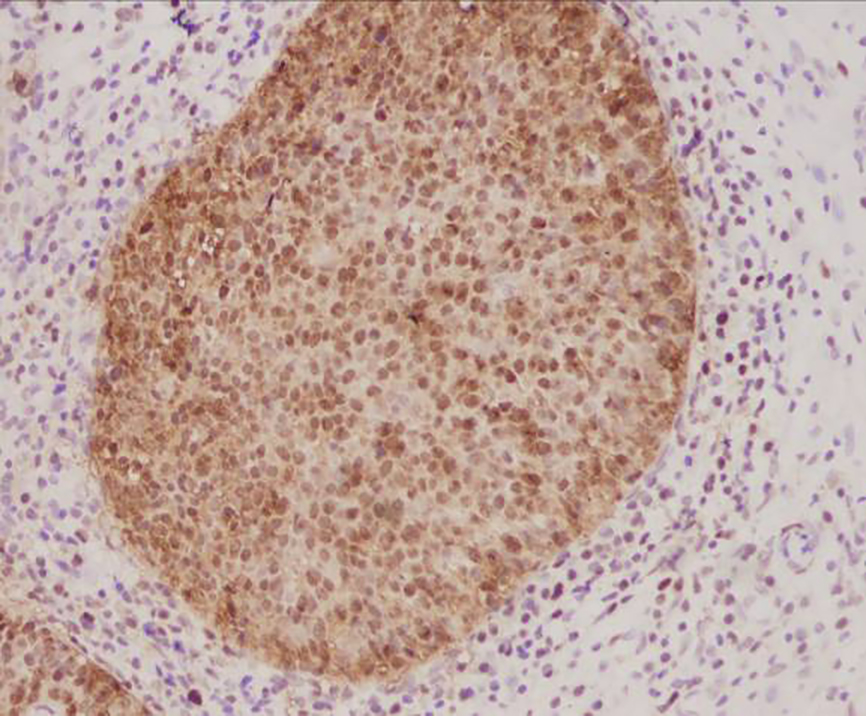

Supplement: Supplementary file 1 — Additional file 1: [file 12885_2023_10543_MOESM1_ESM.zip › Fig1 PCNA protein in cervical cancer tissue 300ppi original.jpg]

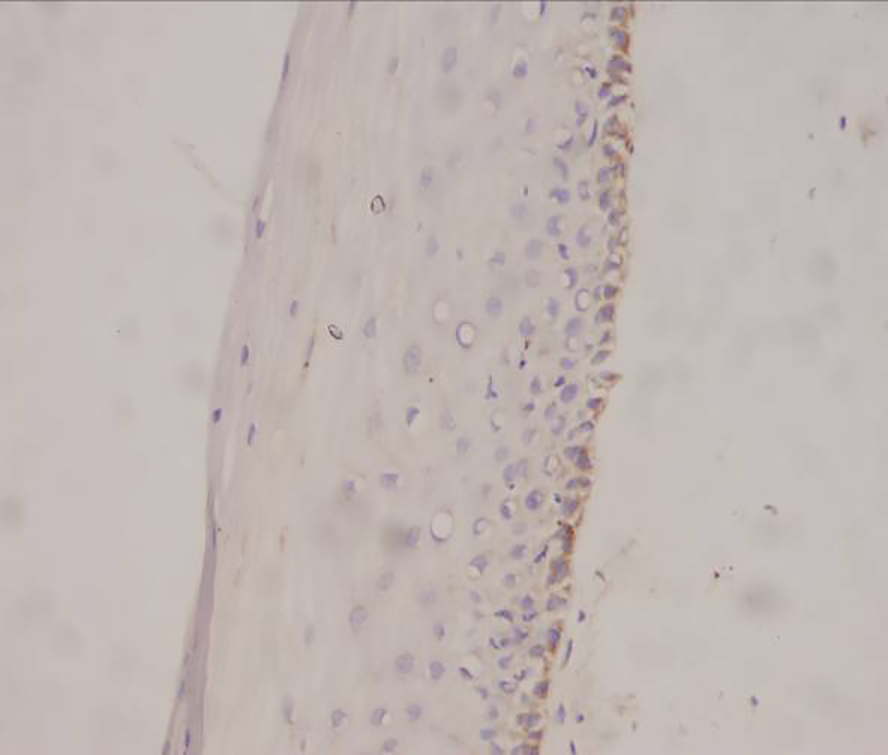

Supplement: Supplementary file 1 — Additional file 1: [file 12885_2023_10543_MOESM1_ESM.zip › Fig1 PCNA protein in chronic cervicitis tissue 300ppi original.jpg]

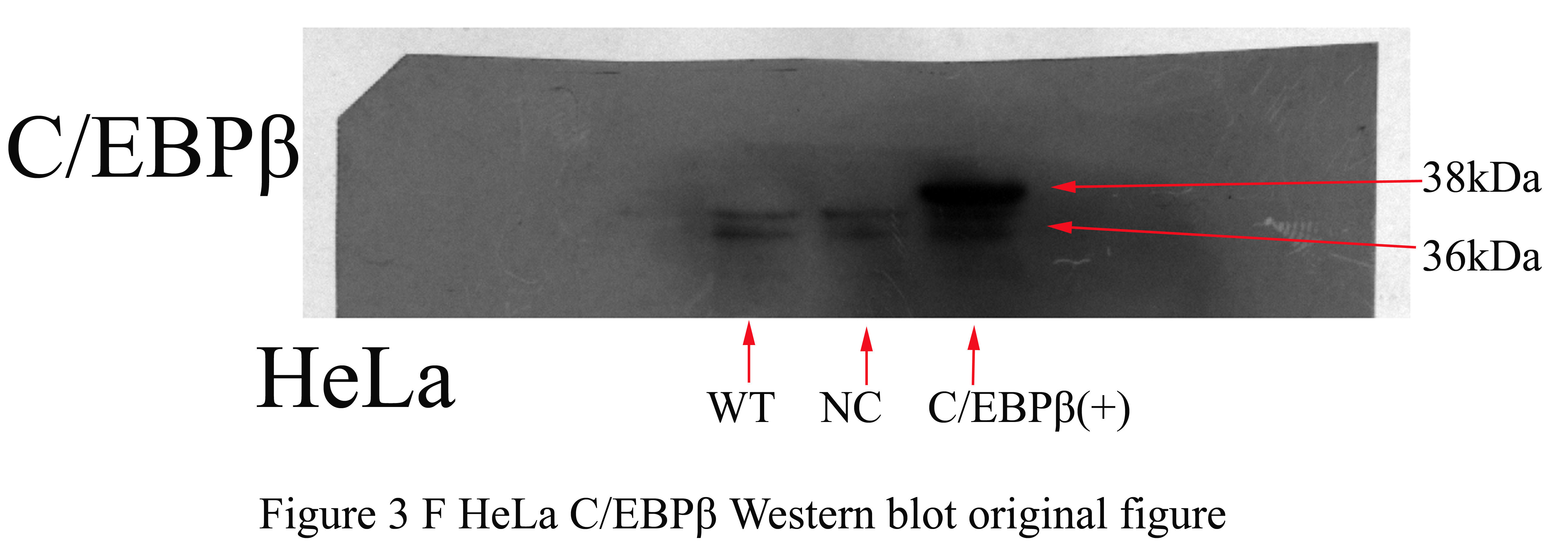

Supplement: Supplementary file 1 — Additional file 1: [file 12885_2023_10543_MOESM1_ESM.zip › Fig3F1 CEBPB in Hela photographic plate.jpg]

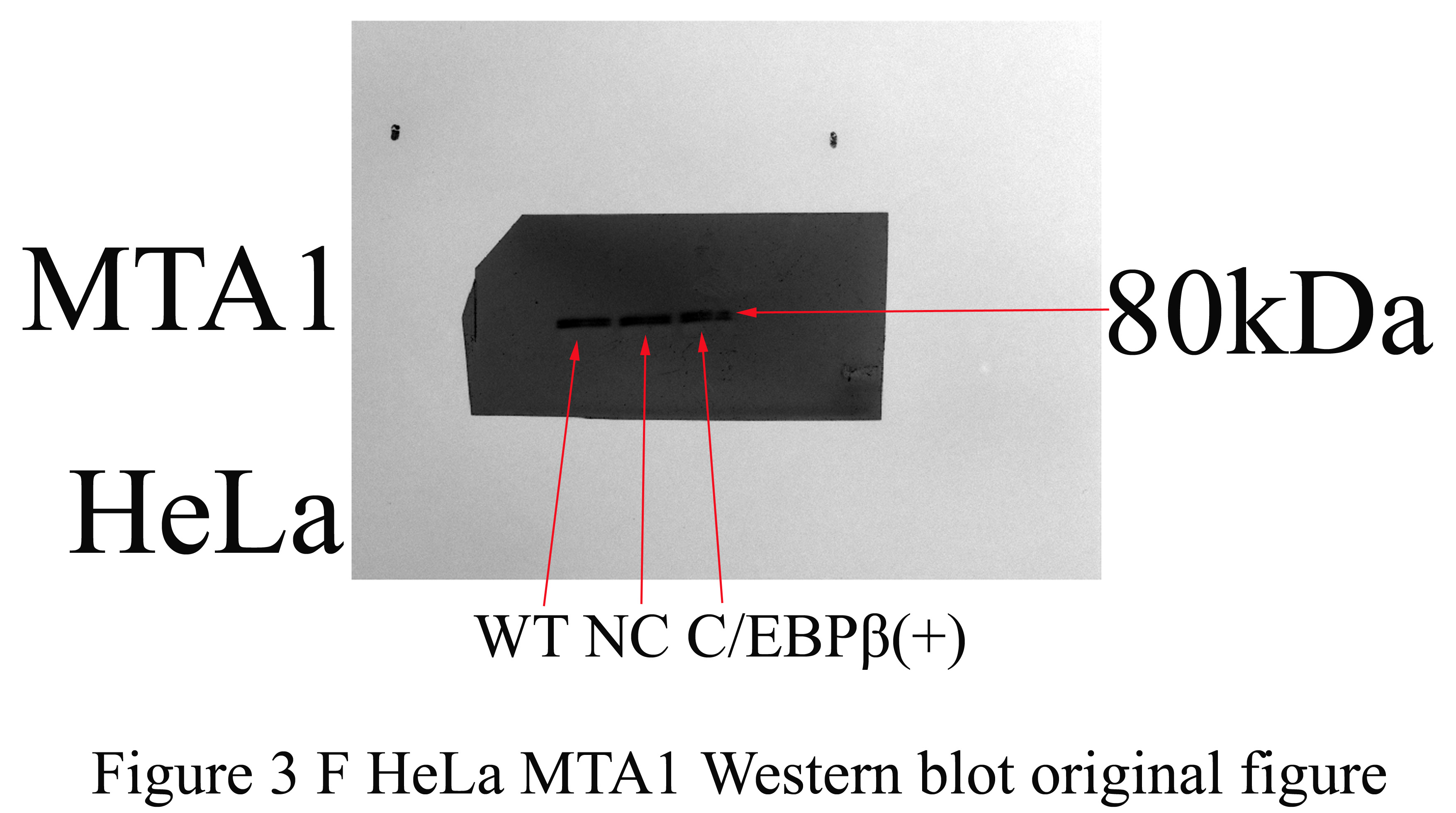

Supplement: Supplementary file 1 — Additional file 1: [file 12885_2023_10543_MOESM1_ESM.zip › Fig3F2 MTA1(WT,NC,CEBPB) photographic plate.jpg]

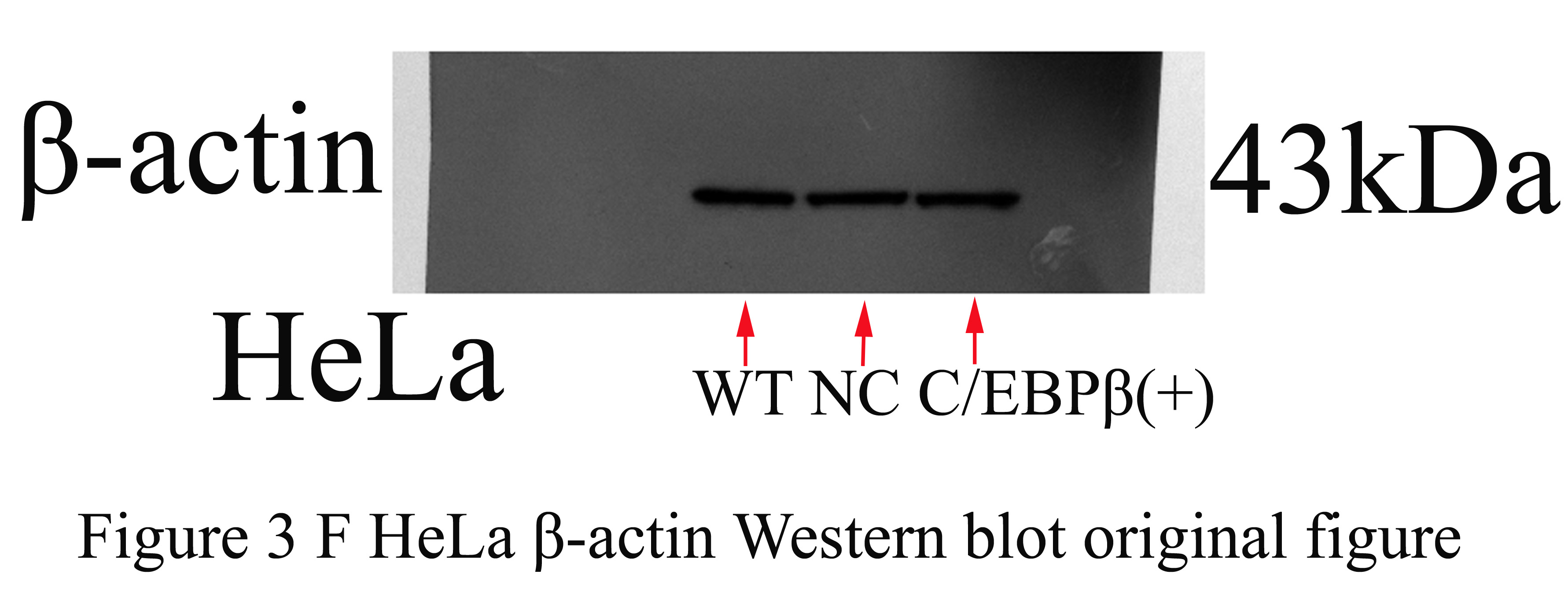

Supplement: Supplementary file 1 — Additional file 1: [file 12885_2023_10543_MOESM1_ESM.zip › Fig3F3 beta-actin photographic plate.jpg]

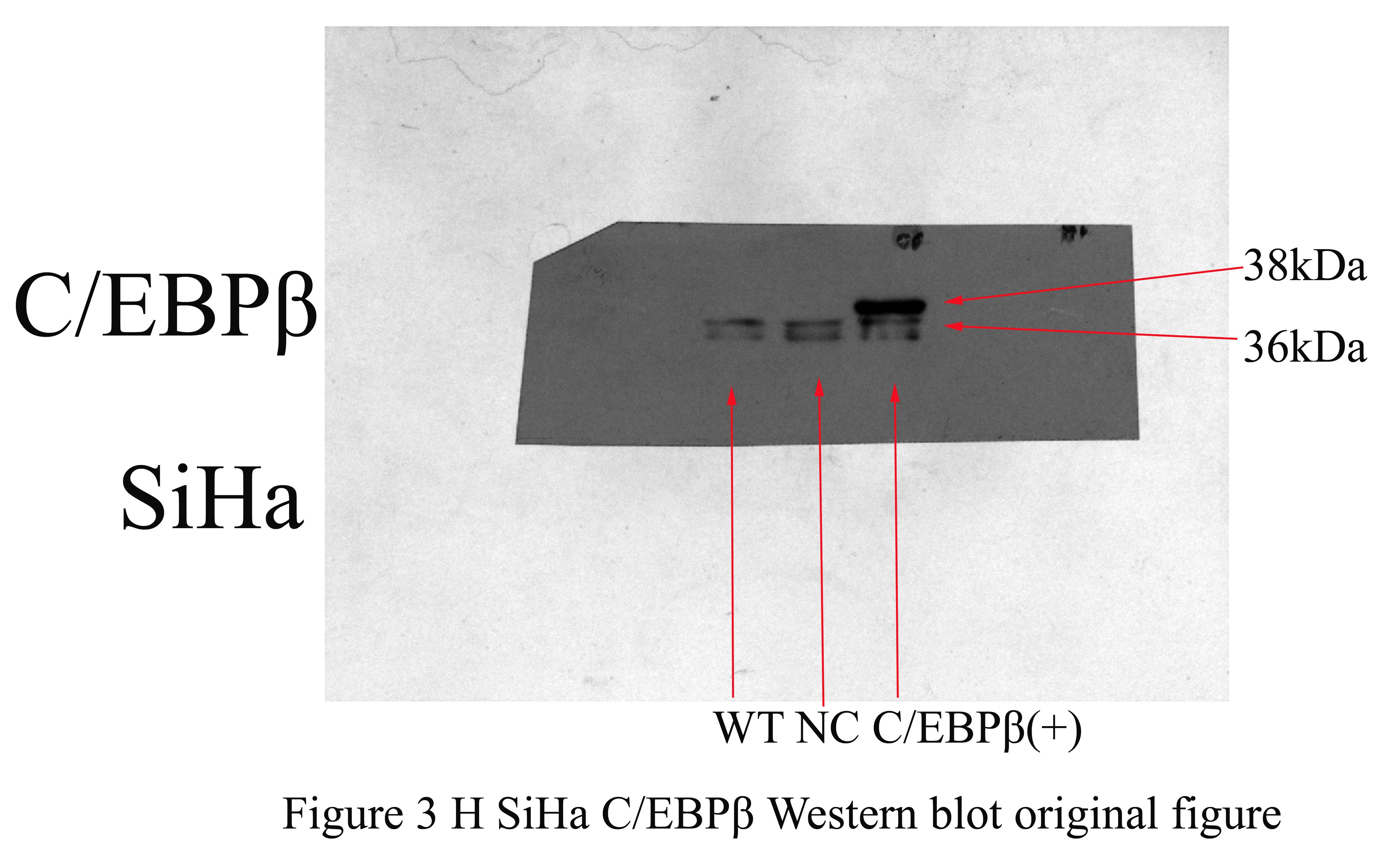

Supplement: Supplementary file 1 — Additional file 1: [file 12885_2023_10543_MOESM1_ESM.zip › Fig3H1 cebpb photographic plate.jpg]

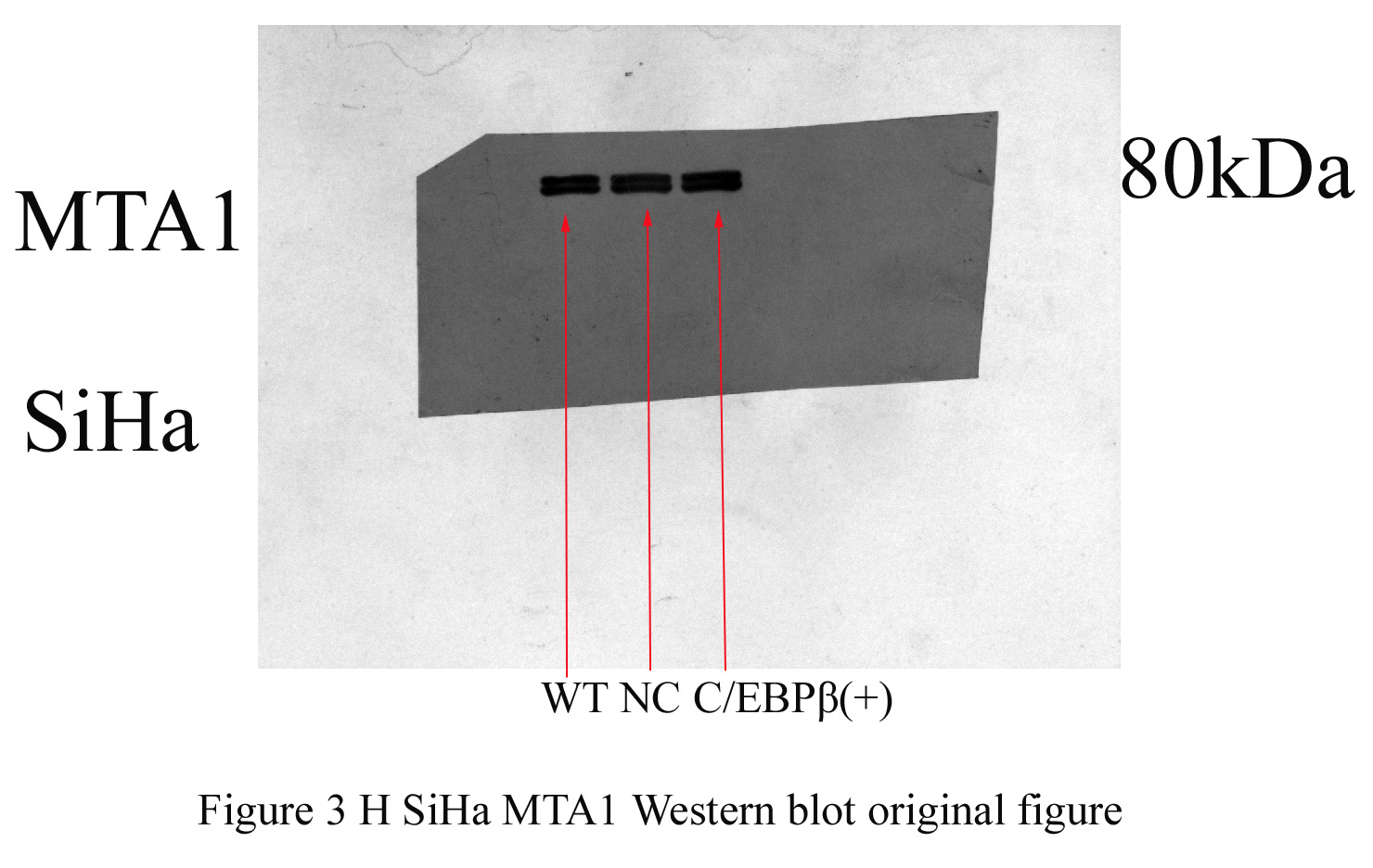

Supplement: Supplementary file 1 — Additional file 1: [file 12885_2023_10543_MOESM1_ESM.zip › Fig3H2 MTA1 photographic plate.jpg]

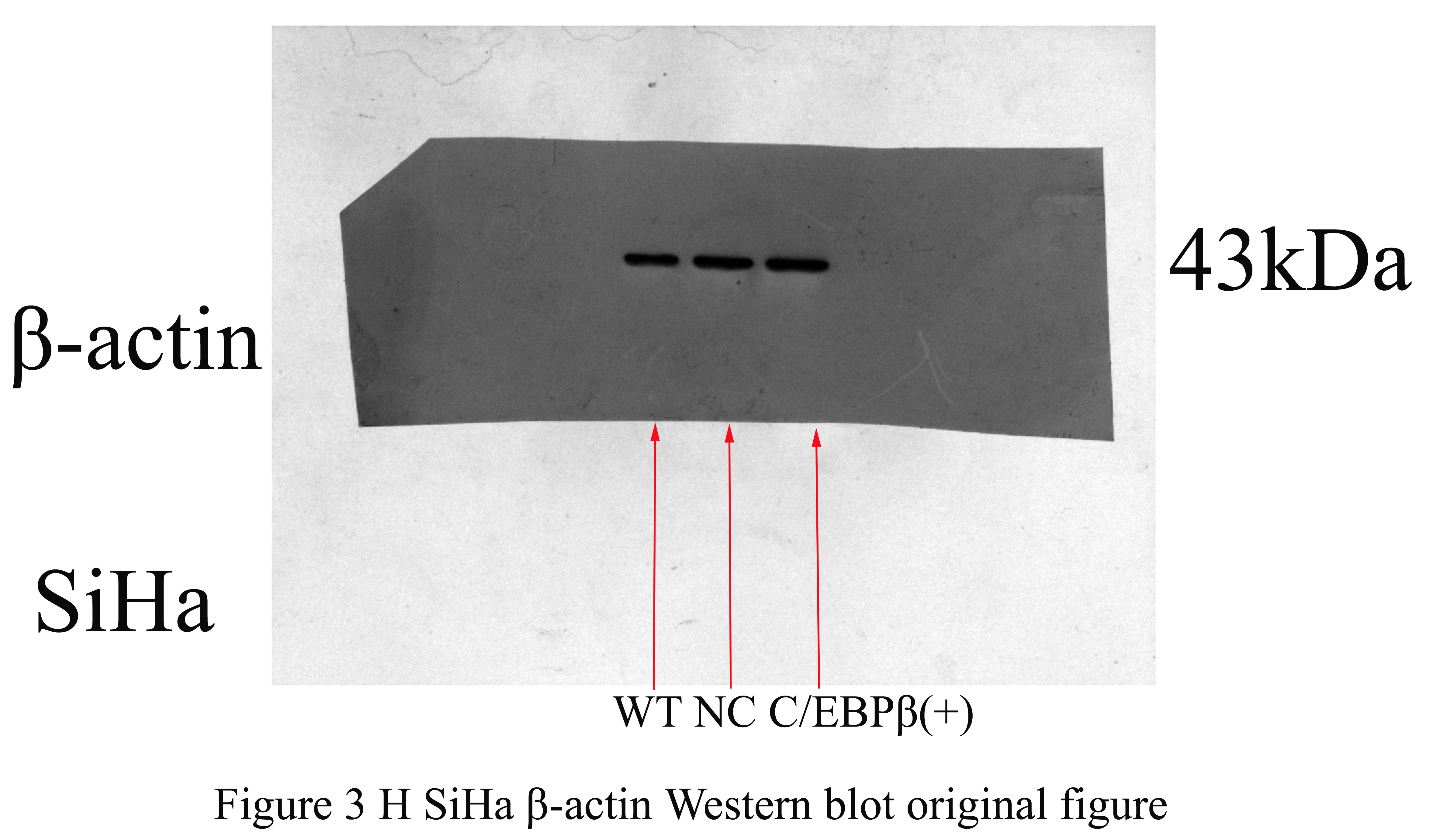

Supplement: Supplementary file 1 — Additional file 1: [file 12885_2023_10543_MOESM1_ESM.zip › Fig3H3 b-actin photographic plate.jpg]

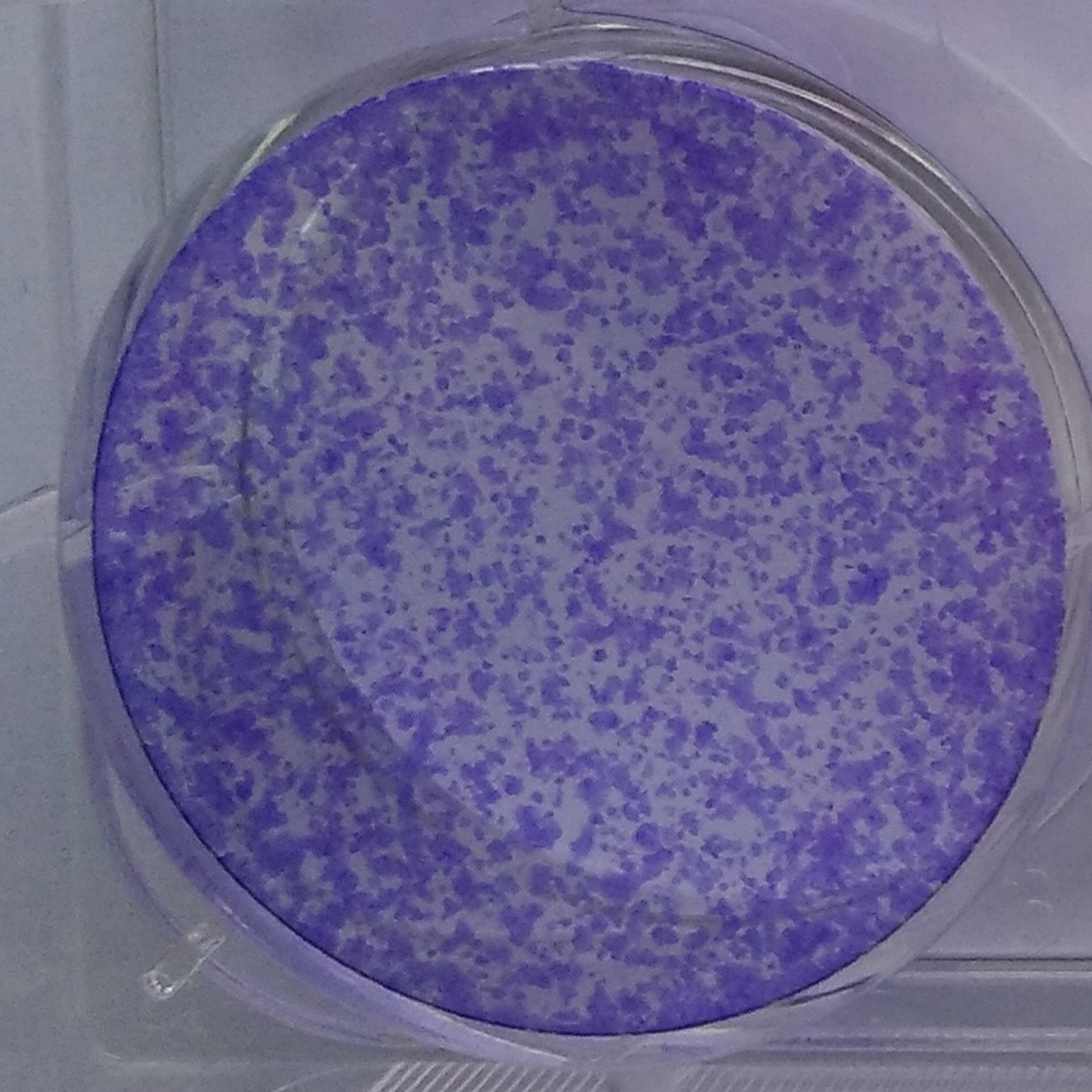

Supplement: Supplementary file 1 — Additional file 1: [file 12885_2023_10543_MOESM1_ESM.zip › Fig4C hela-NC.jpg]

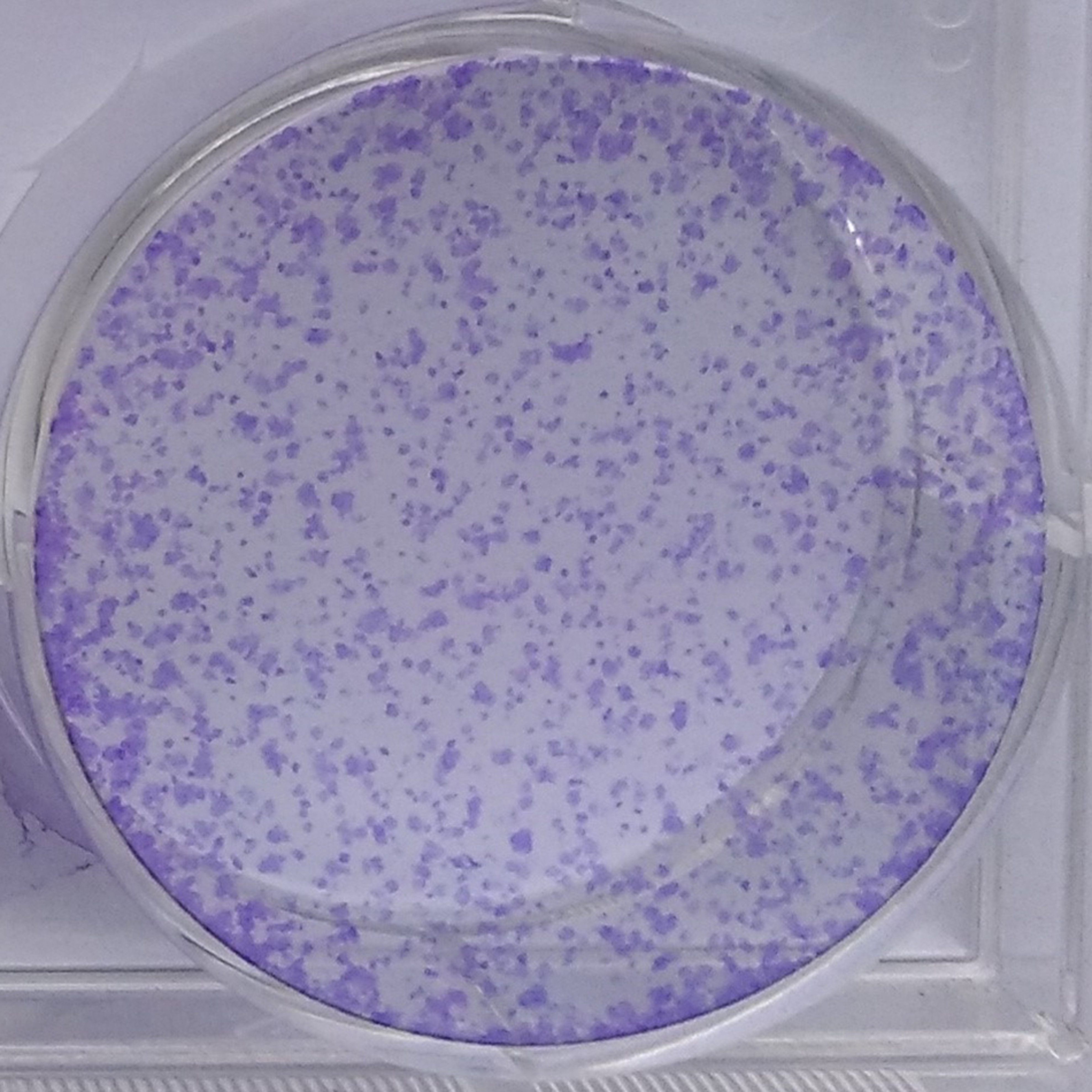

Supplement: Supplementary file 1 — Additional file 1: [file 12885_2023_10543_MOESM1_ESM.zip › Fig4D hela-CEBPB.jpg]

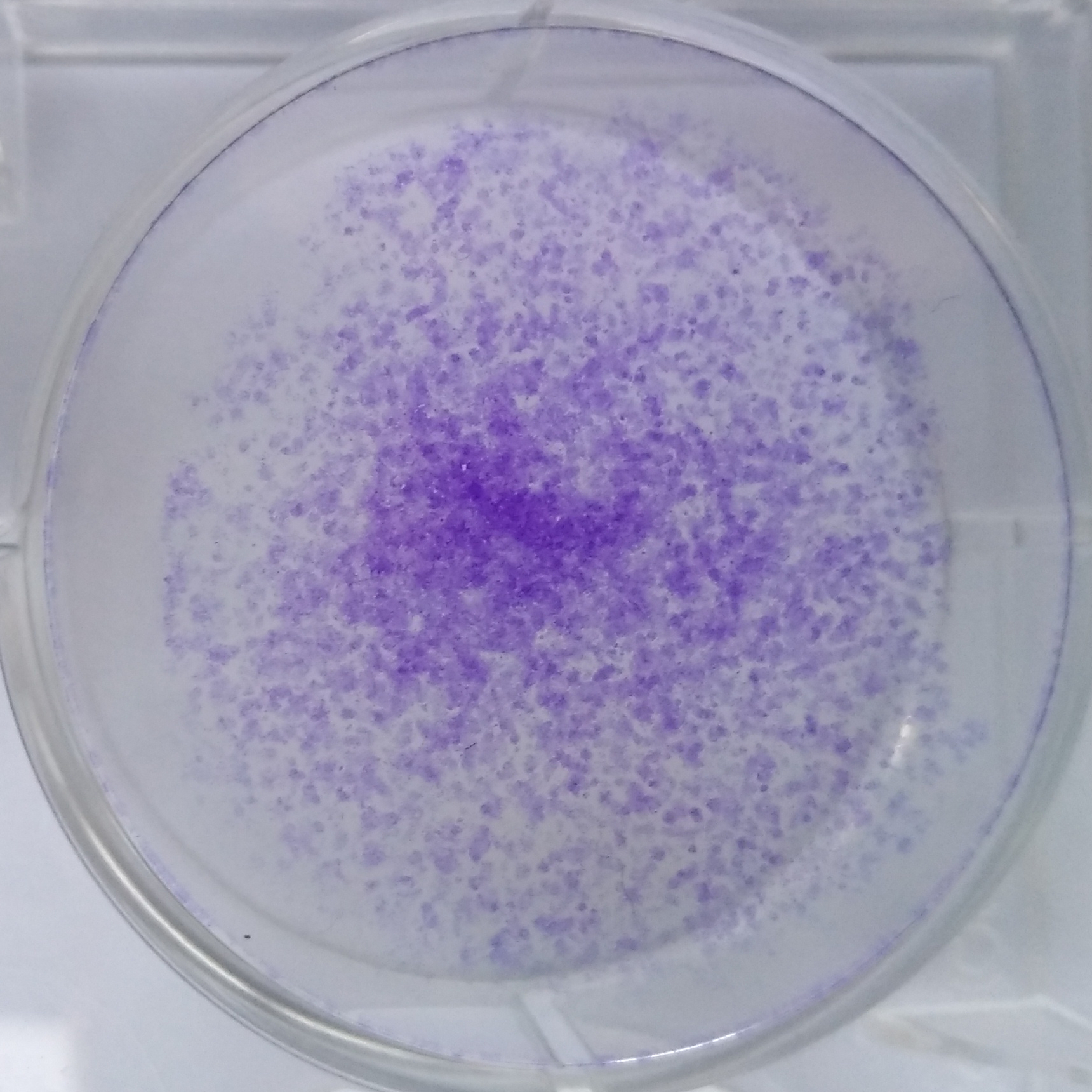

Supplement: Supplementary file 1 — Additional file 1: [file 12885_2023_10543_MOESM1_ESM.zip › Fig4F siha-NC.jpg]

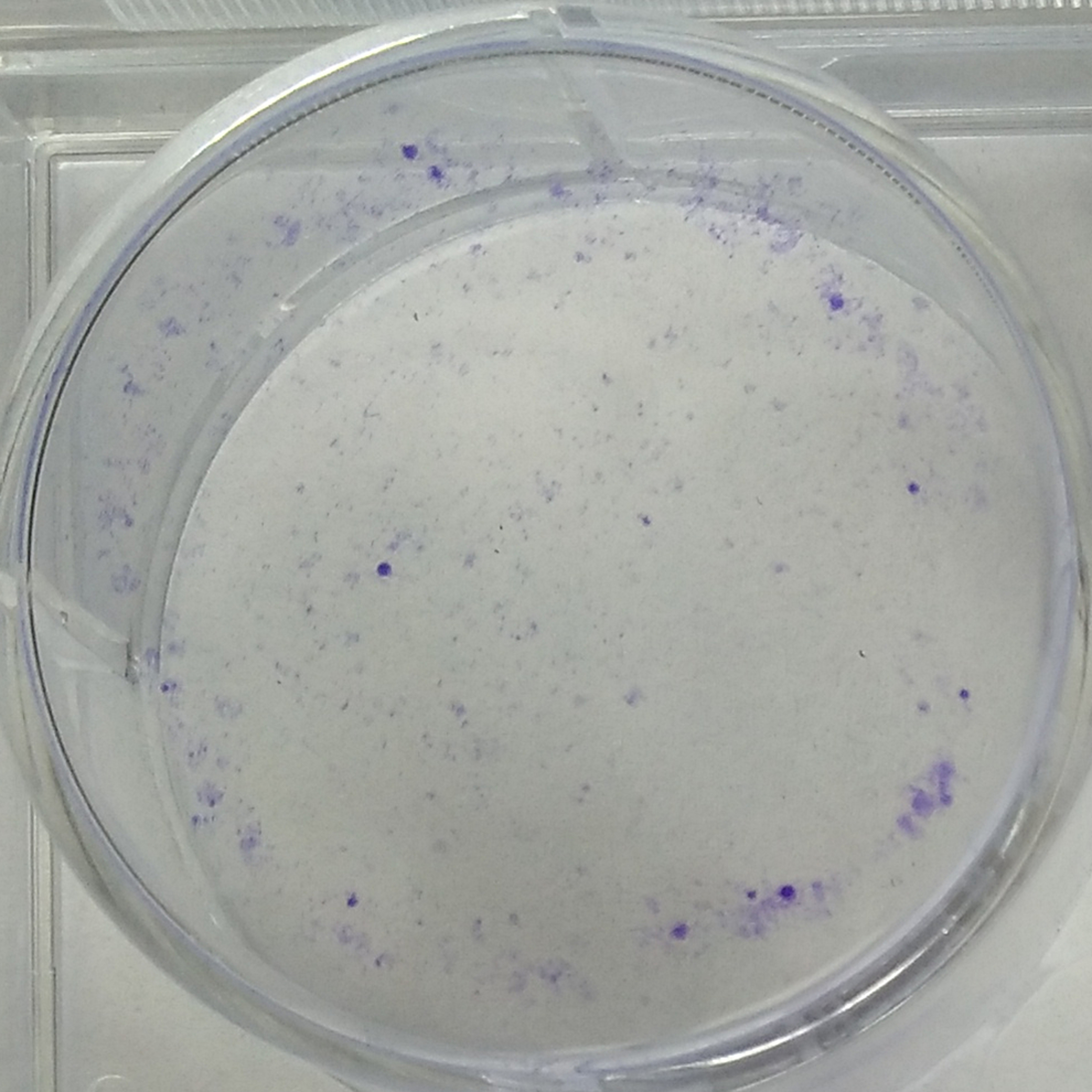

Supplement: Supplementary file 1 — Additional file 1: [file 12885_2023_10543_MOESM1_ESM.zip › Fig4G siha-CEBPB.jpg]

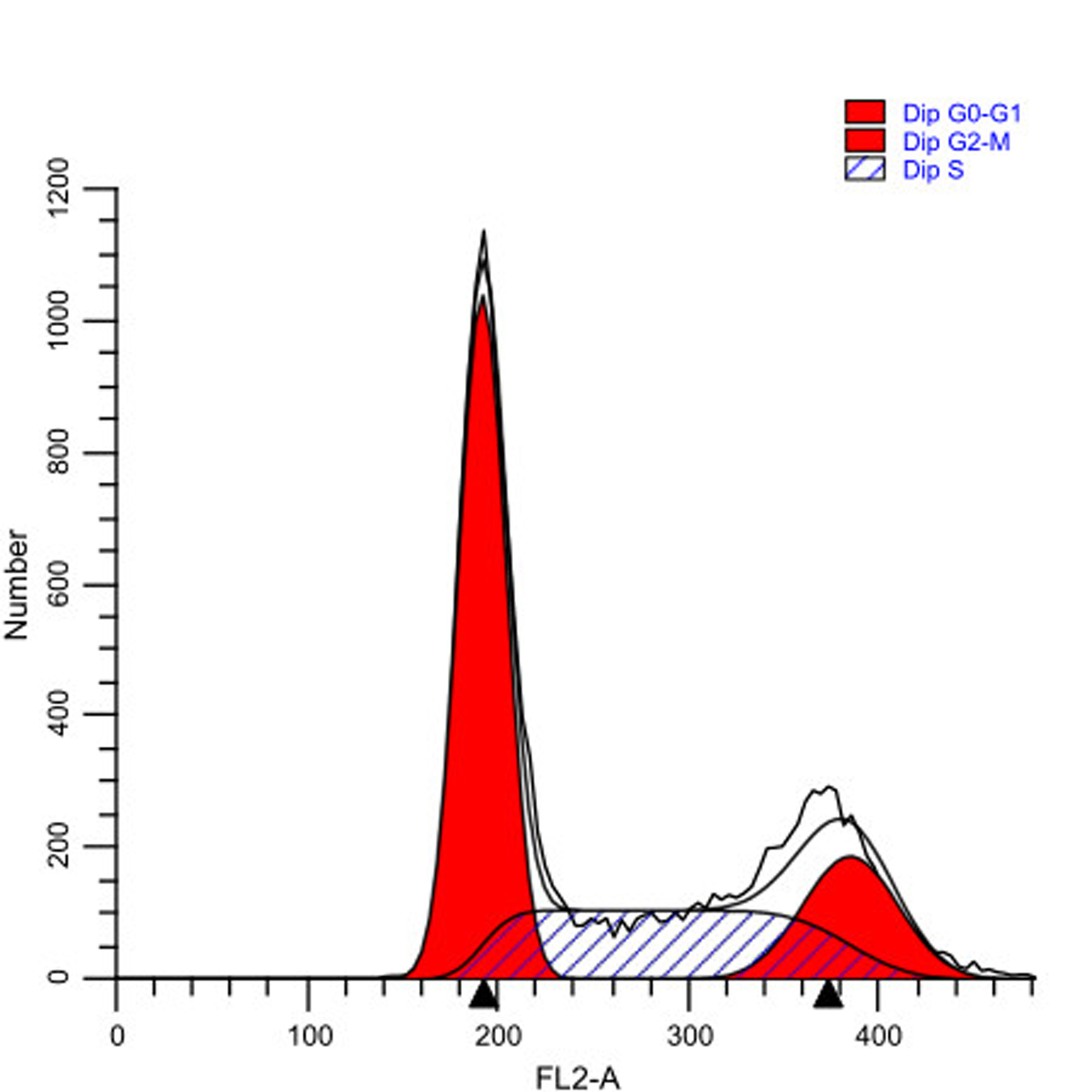

Supplement: Supplementary file 1 — Additional file 1: [file 12885_2023_10543_MOESM1_ESM.zip › Fig4I Hela-NC.jpg]

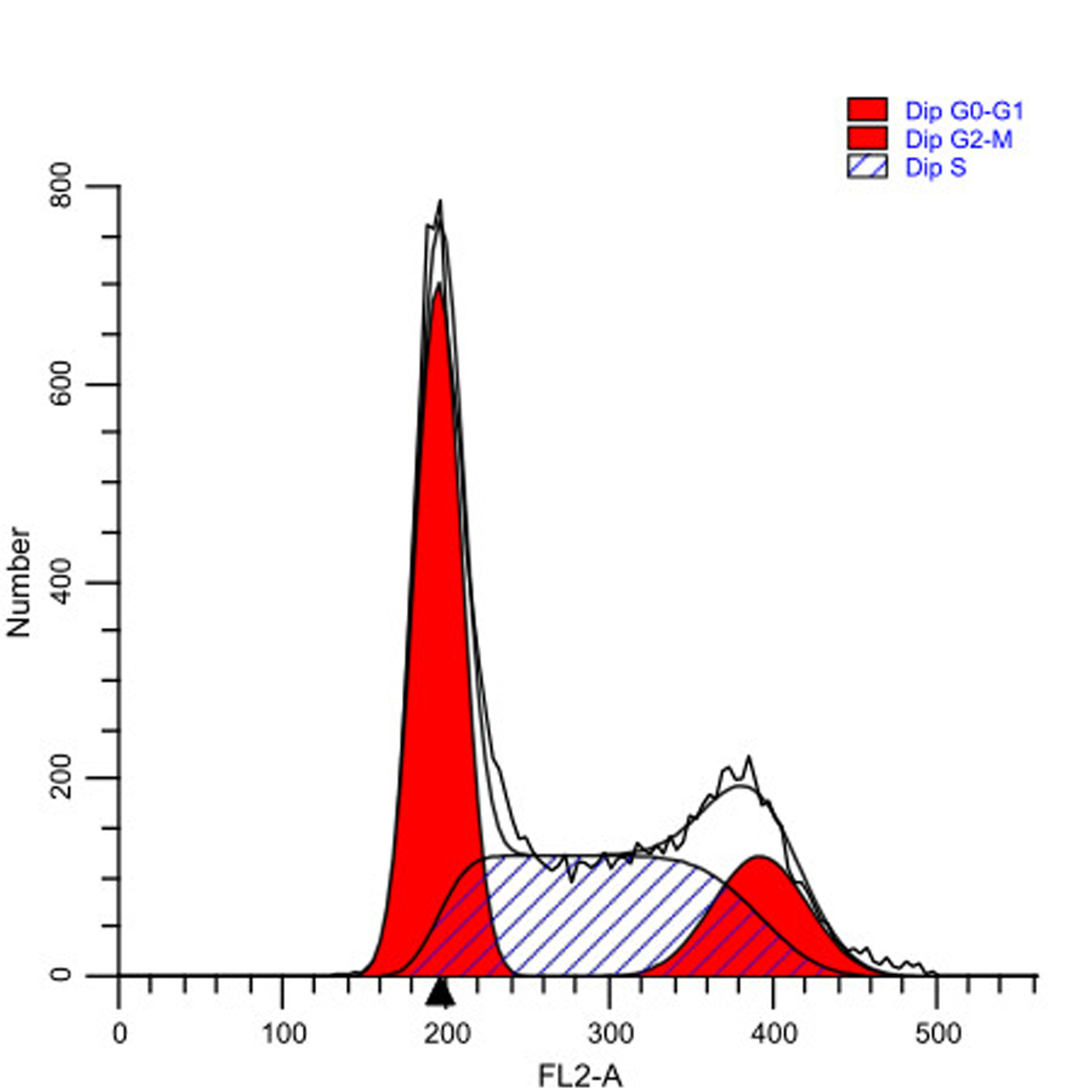

Supplement: Supplementary file 1 — Additional file 1: [file 12885_2023_10543_MOESM1_ESM.zip › Fig4J Hela-CEBPB+.jpg]

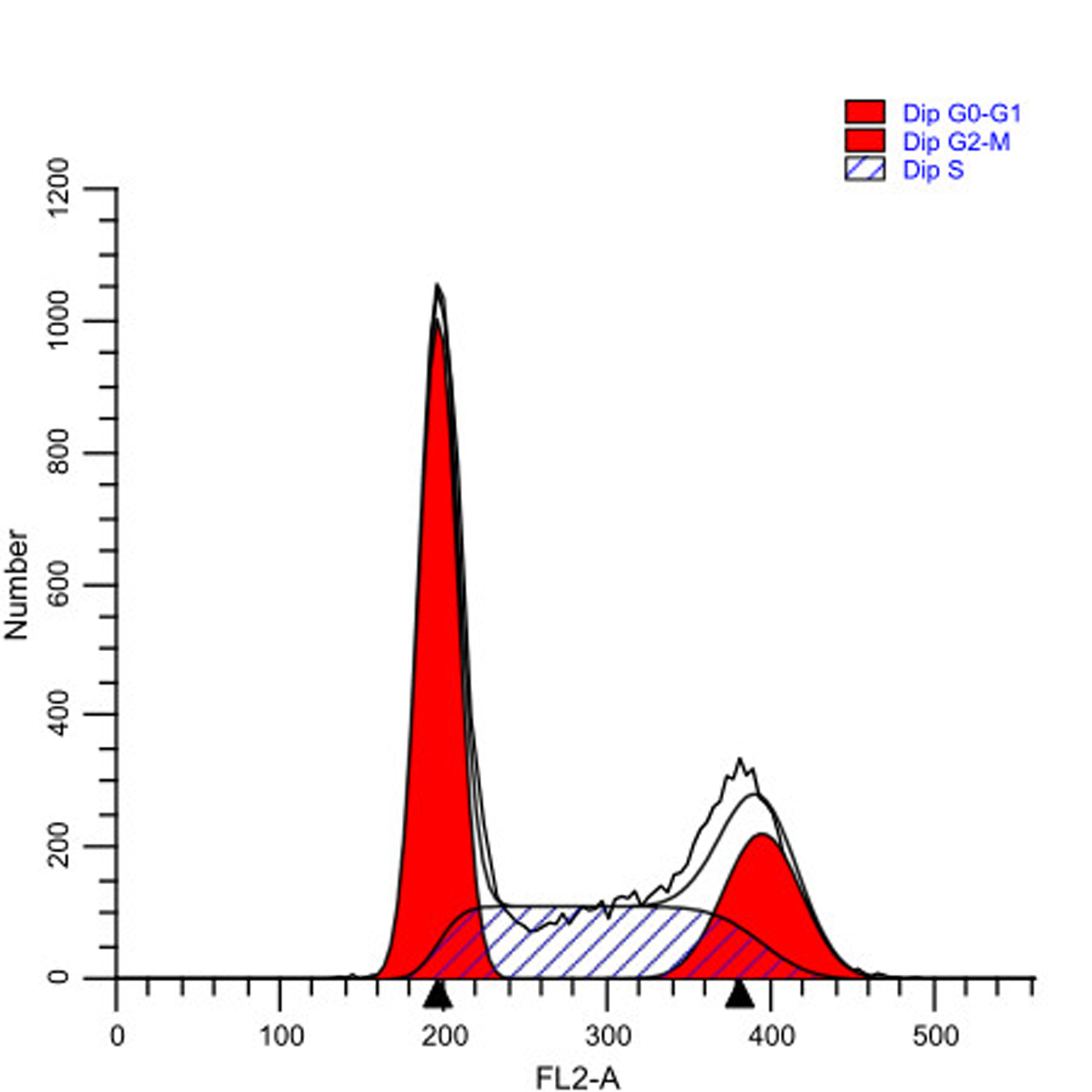

Supplement: Supplementary file 1 — Additional file 1: [file 12885_2023_10543_MOESM1_ESM.zip › Fig4L SiHa-NC.jpg]

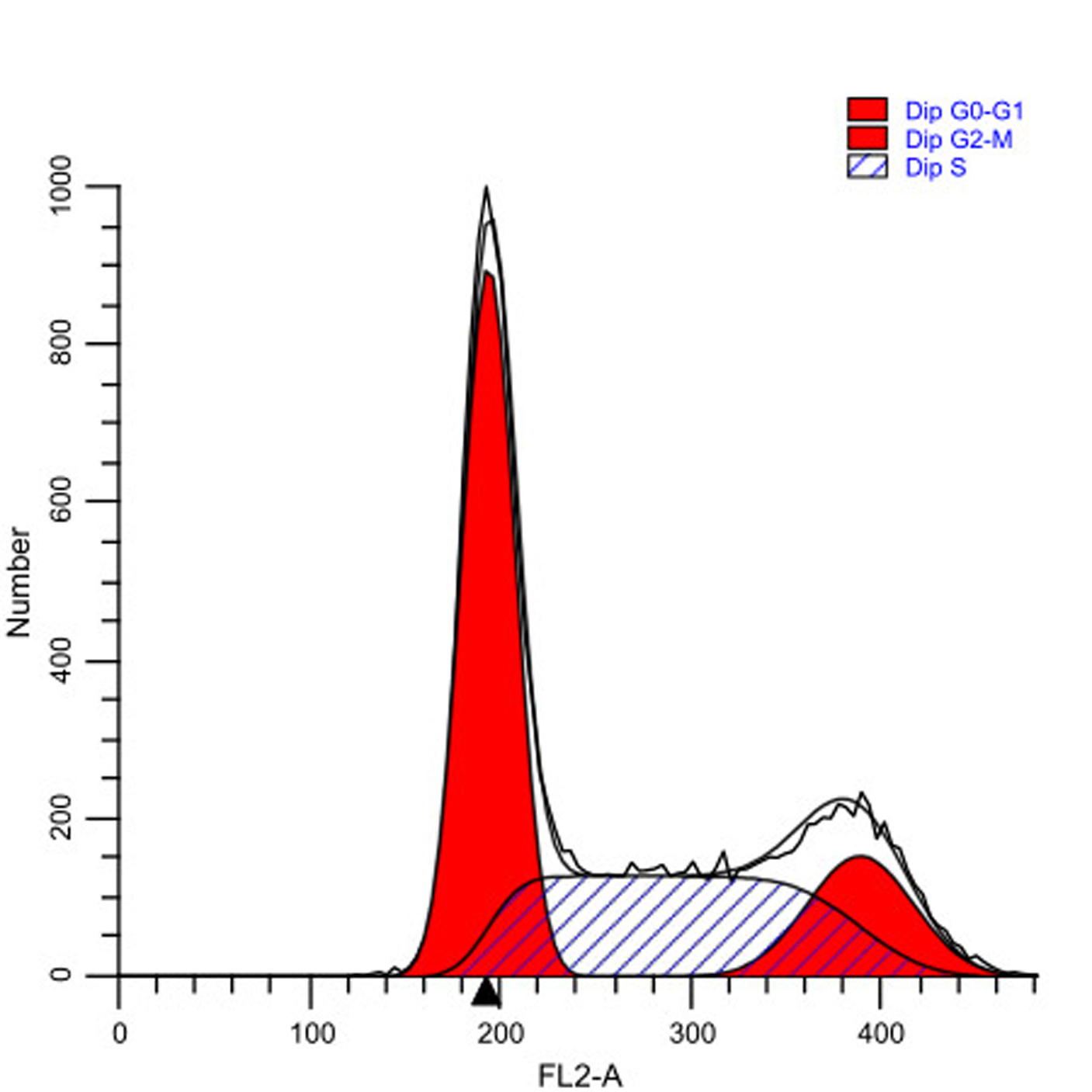

Supplement: Supplementary file 1 — Additional file 1: [file 12885_2023_10543_MOESM1_ESM.zip › Fig4M SiHa-CEBPB+.jpg]

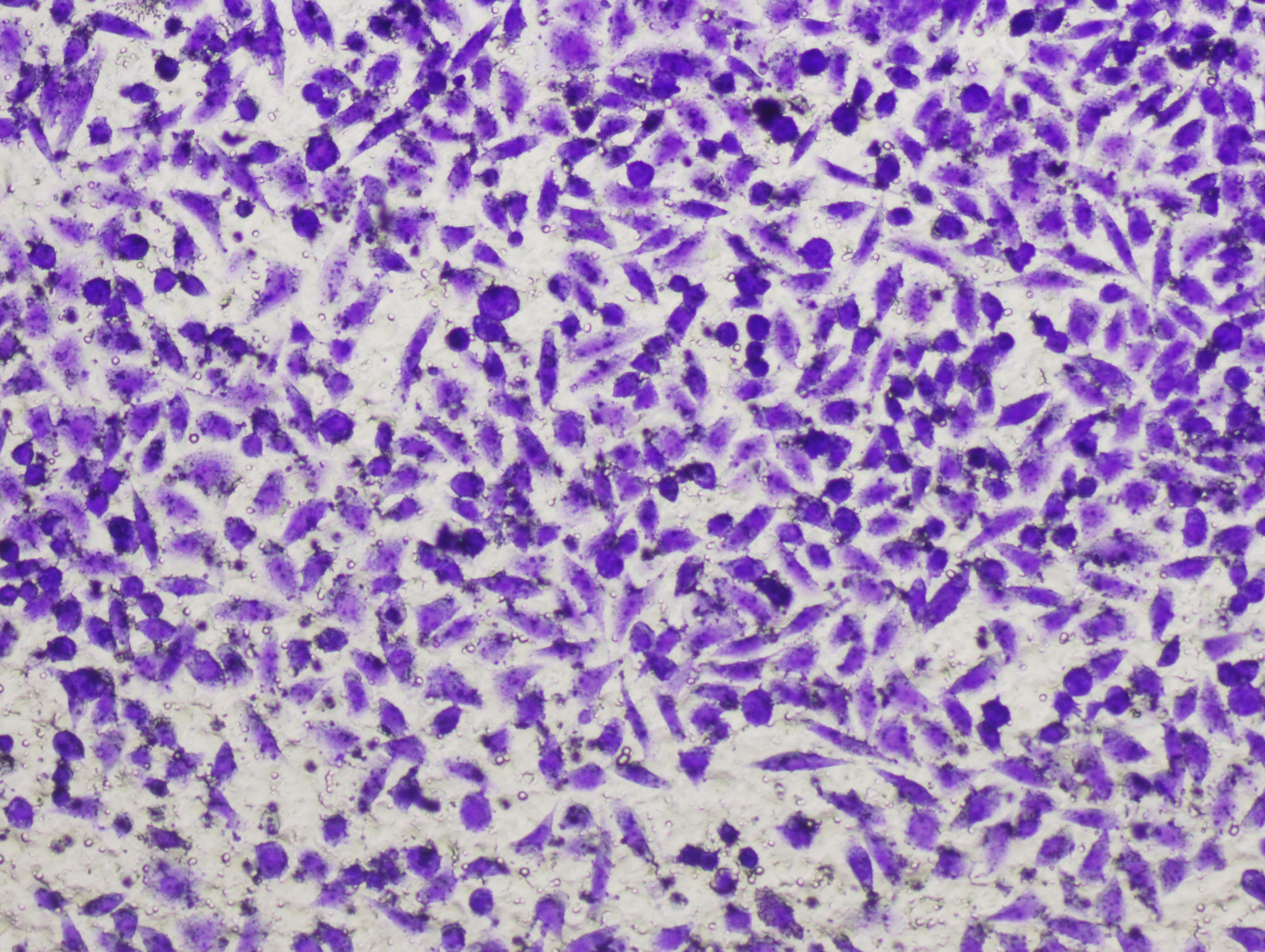

Supplement: Supplementary file 1 — Additional file 1: [file 12885_2023_10543_MOESM1_ESM.zip › Fig5G HeLa NC original.png]

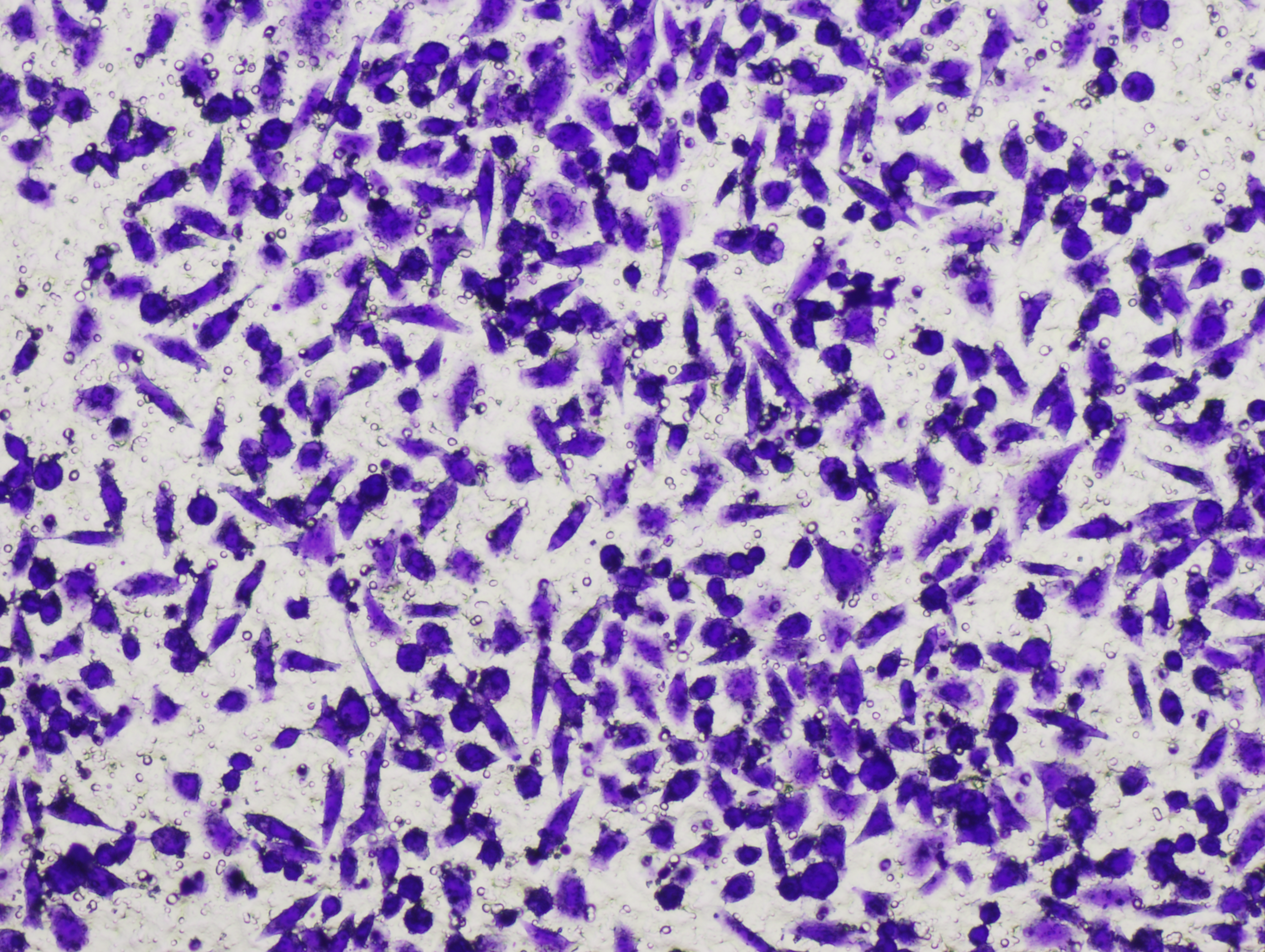

Supplement: Supplementary file 1 — Additional file 1: [file 12885_2023_10543_MOESM1_ESM.zip › Fig5H HeLa CEBPB+ original.png]

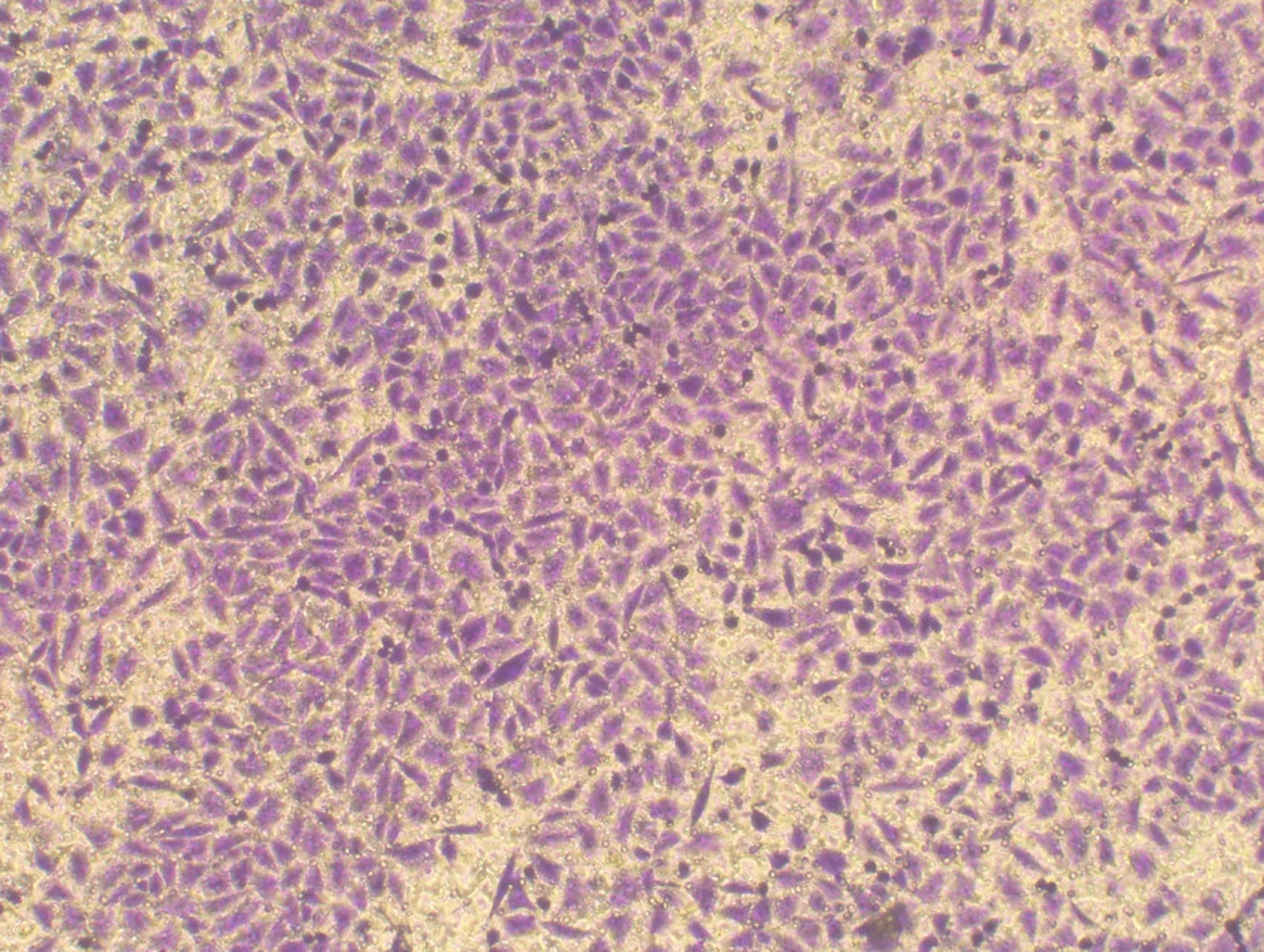

Supplement: Supplementary file 1 — Additional file 1: [file 12885_2023_10543_MOESM1_ESM.zip › Fig5J SiHa NC original.png]

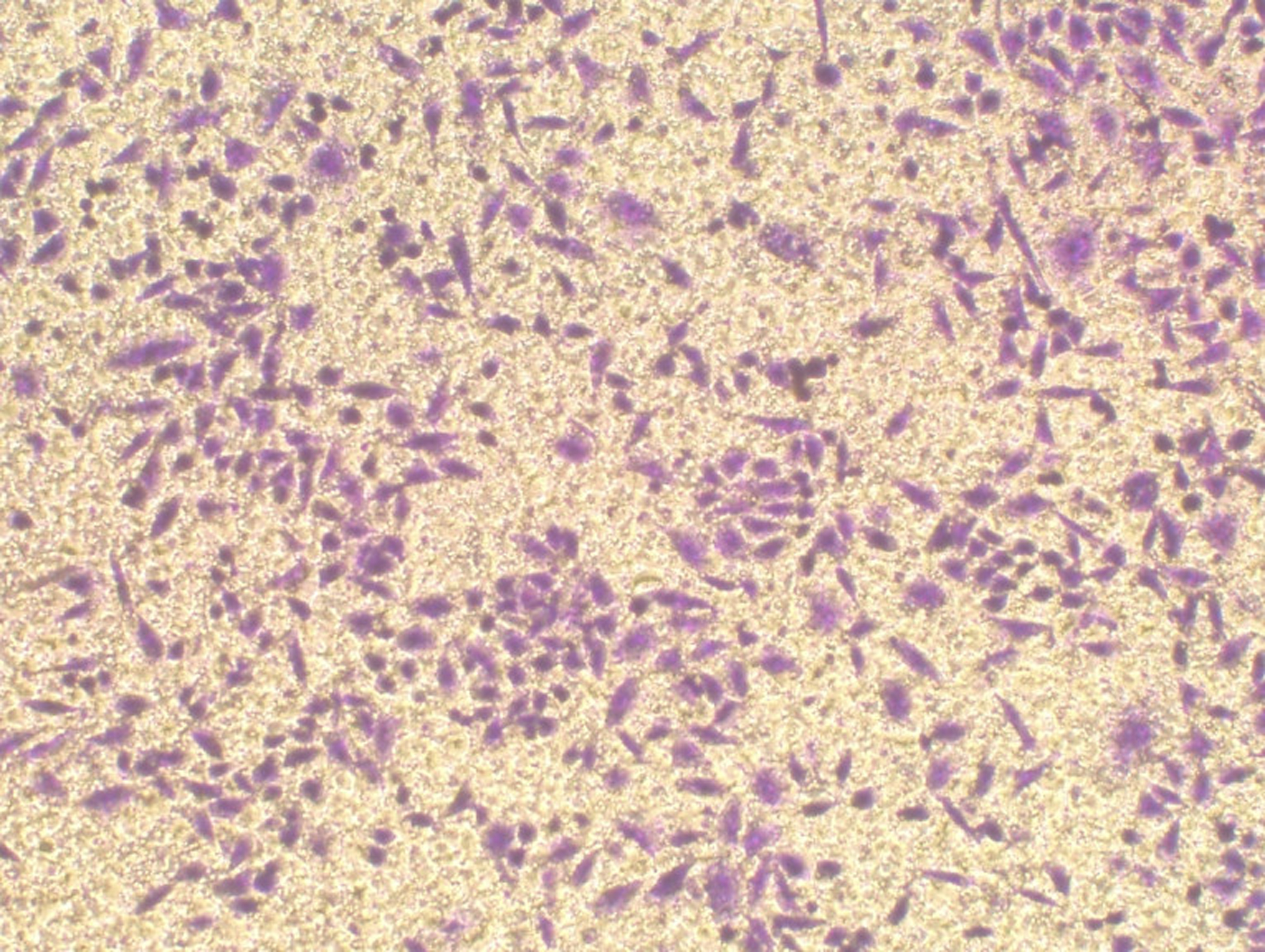

Supplement: Supplementary file 1 — Additional file 1: [file 12885_2023_10543_MOESM1_ESM.zip › Fig5K SiHa CEBPB+ original.png]

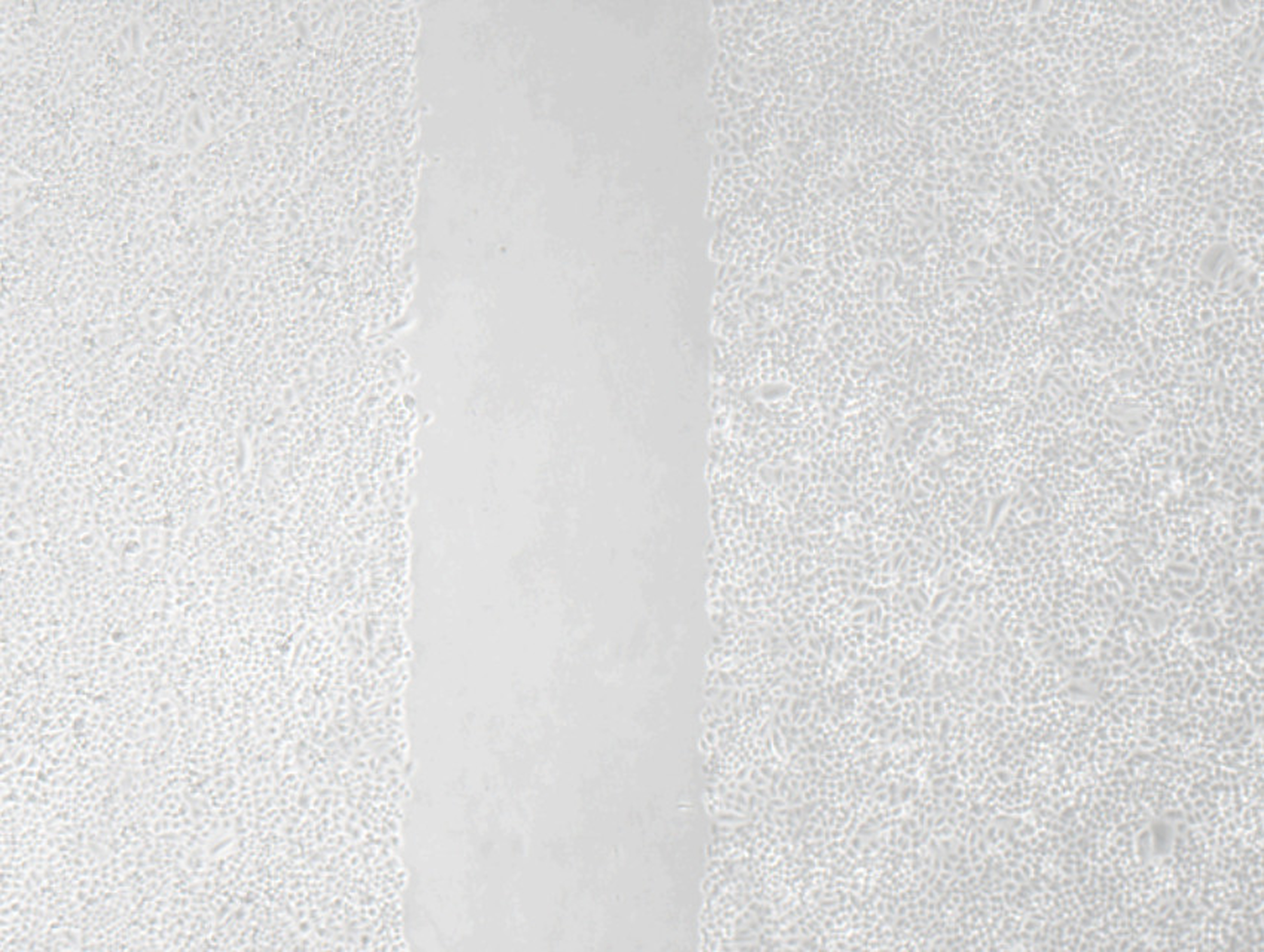

Supplement: Supplementary file 1 — Additional file 1: [file 12885_2023_10543_MOESM1_ESM.zip › Fig6A SiHa CEBPB0h.jpg]

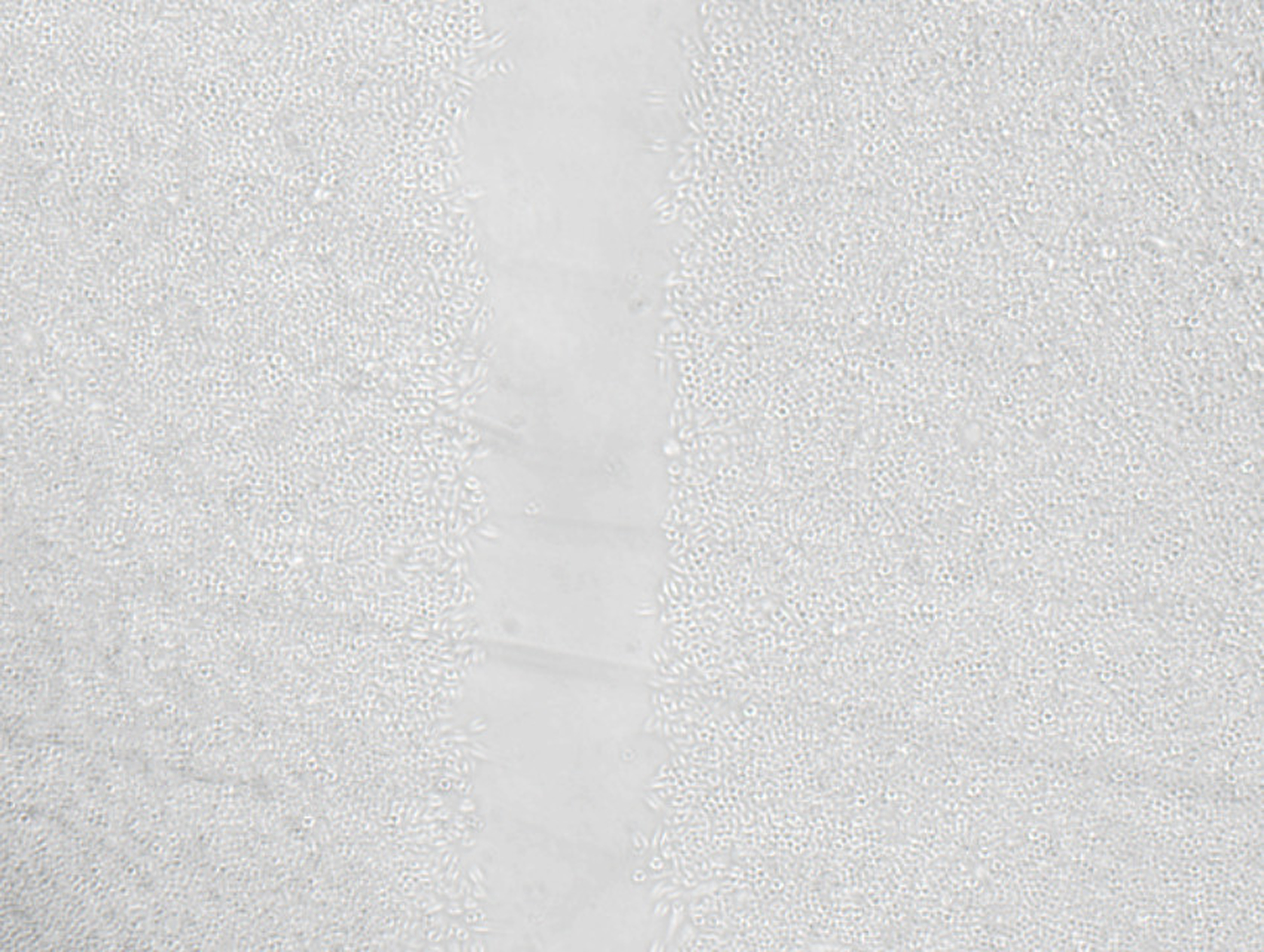

Supplement: Supplementary file 1 — Additional file 1: [file 12885_2023_10543_MOESM1_ESM.zip › Fig6A SiHa CEBPB24h.jpg]

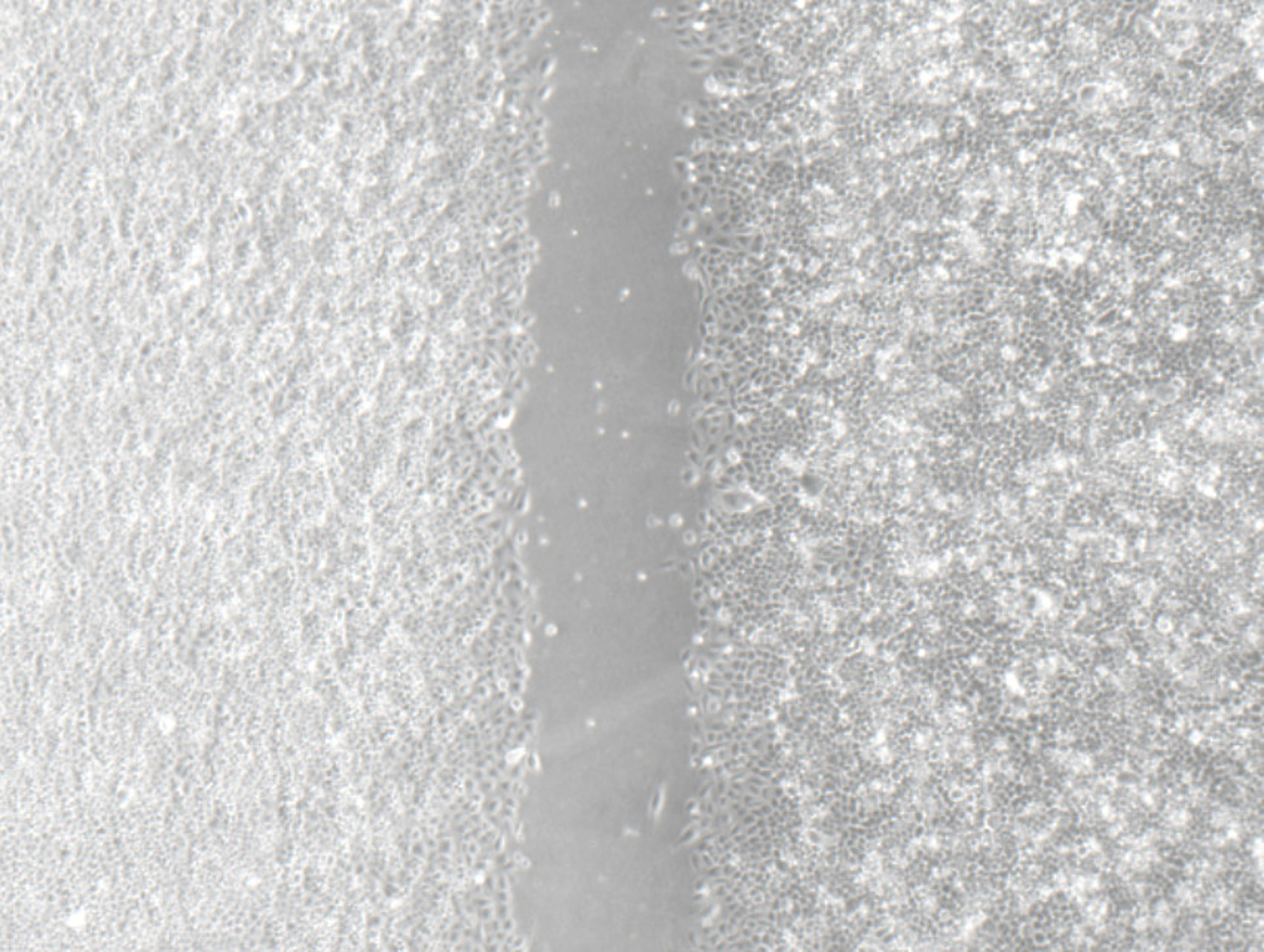

Supplement: Supplementary file 1 — Additional file 1: [file 12885_2023_10543_MOESM1_ESM.zip › Fig6A SiHa CEBPB48h.jpg]

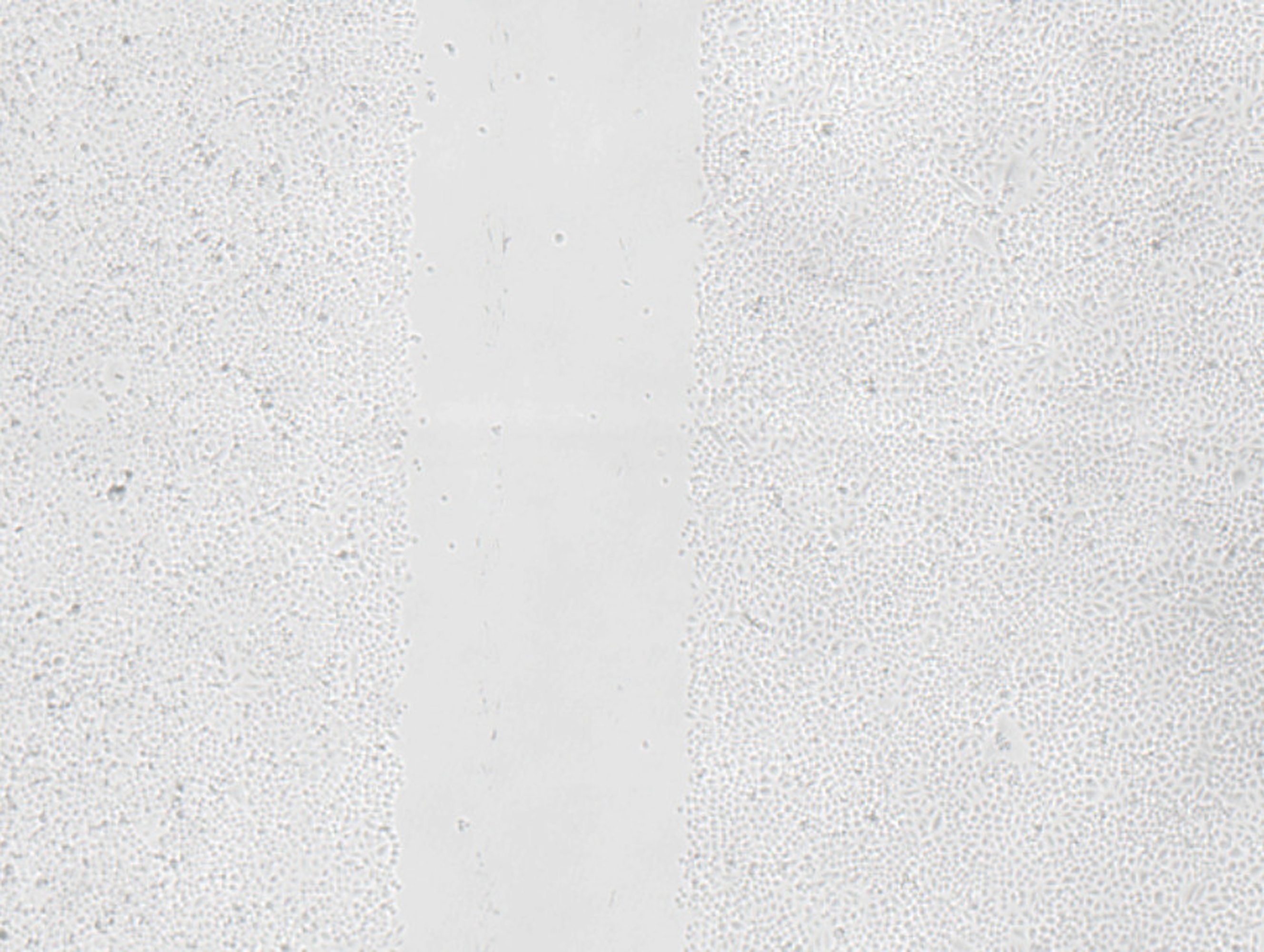

Supplement: Supplementary file 1 — Additional file 1: [file 12885_2023_10543_MOESM1_ESM.zip › Fig6A SiHa NC0h.jpg]

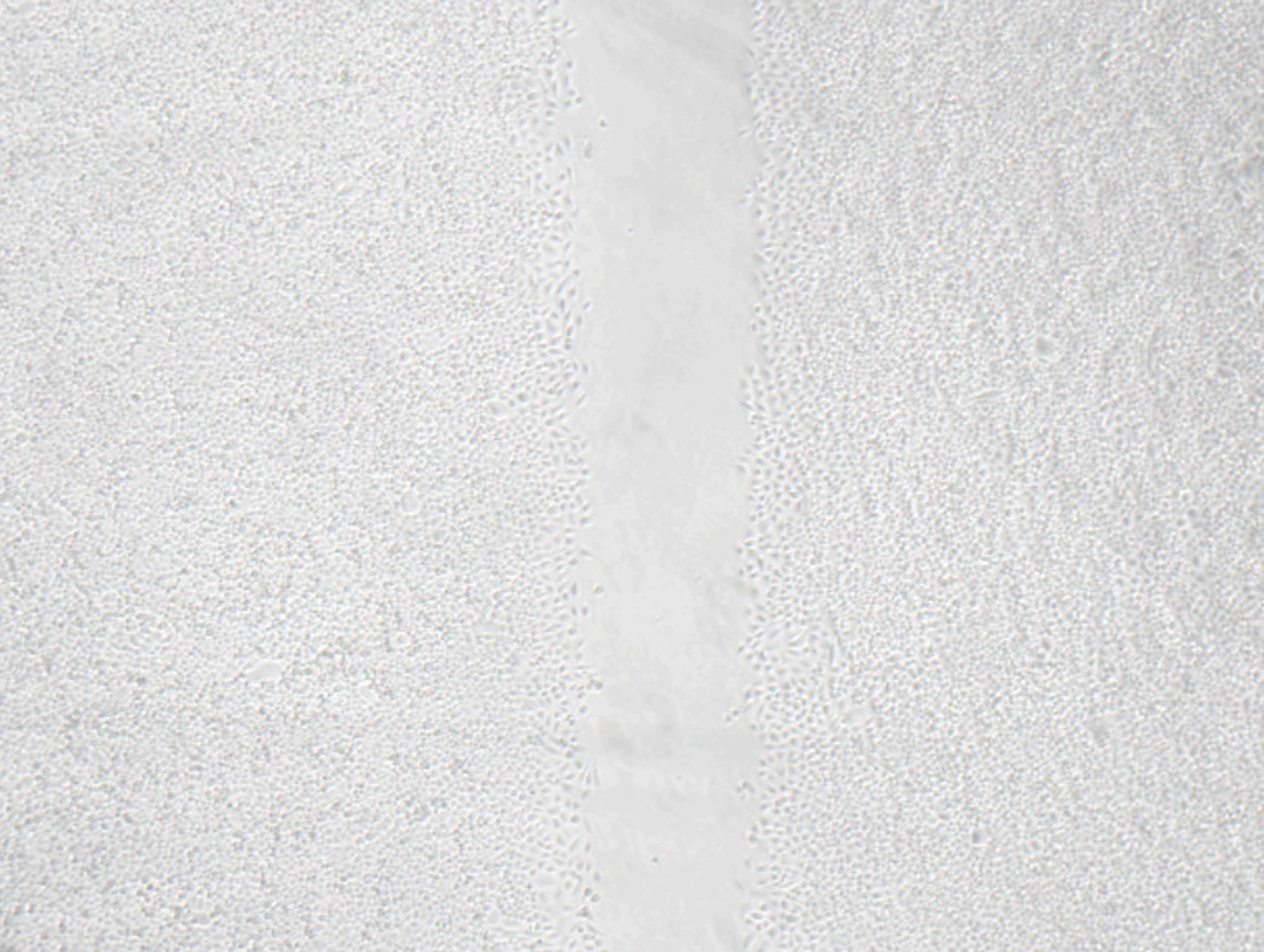

Supplement: Supplementary file 1 — Additional file 1: [file 12885_2023_10543_MOESM1_ESM.zip › Fig6A SiHa NC24h.jpg]

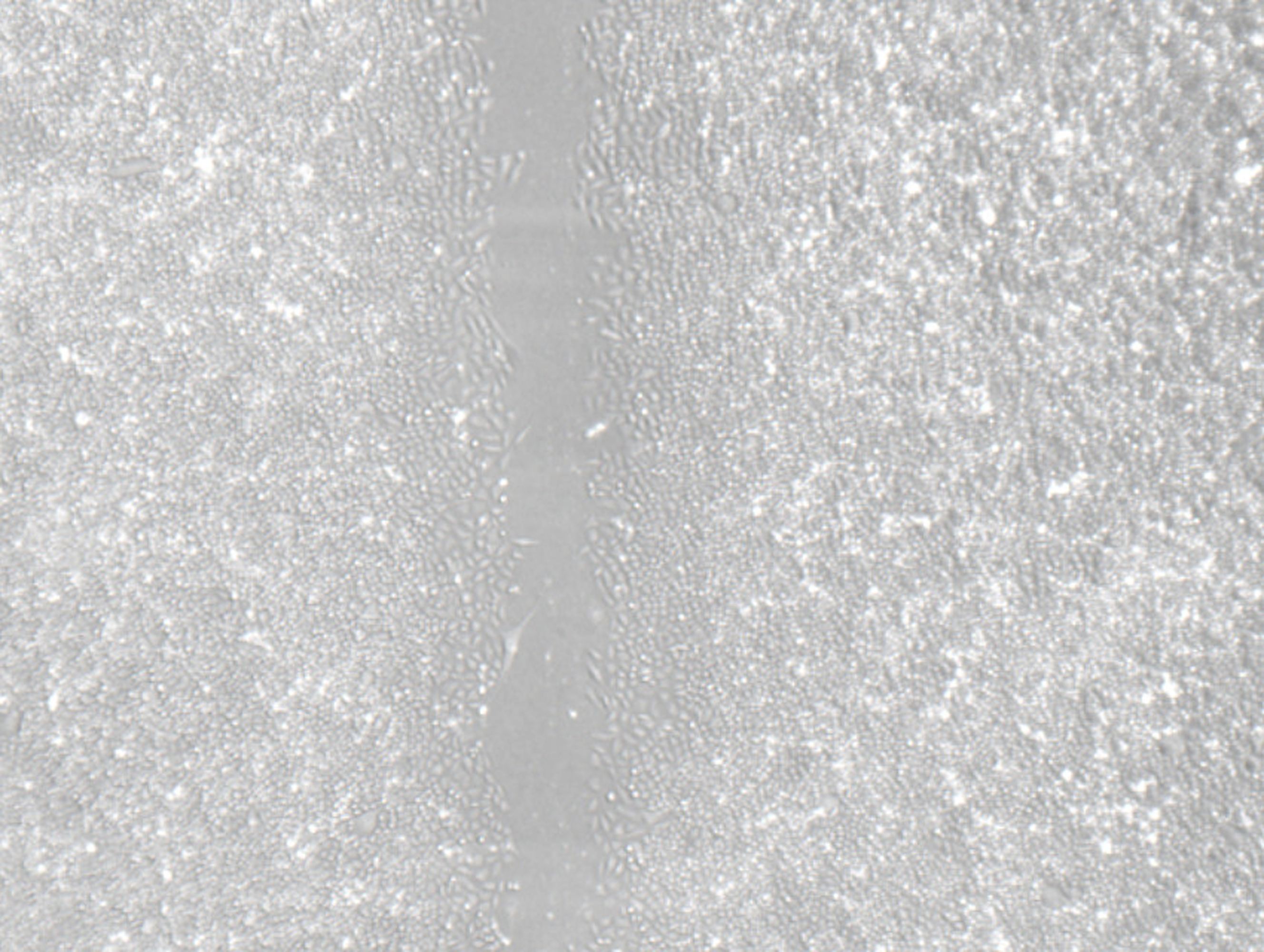

Supplement: Supplementary file 1 — Additional file 1: [file 12885_2023_10543_MOESM1_ESM.zip › Fig6A SiHa NC48h.jpg]

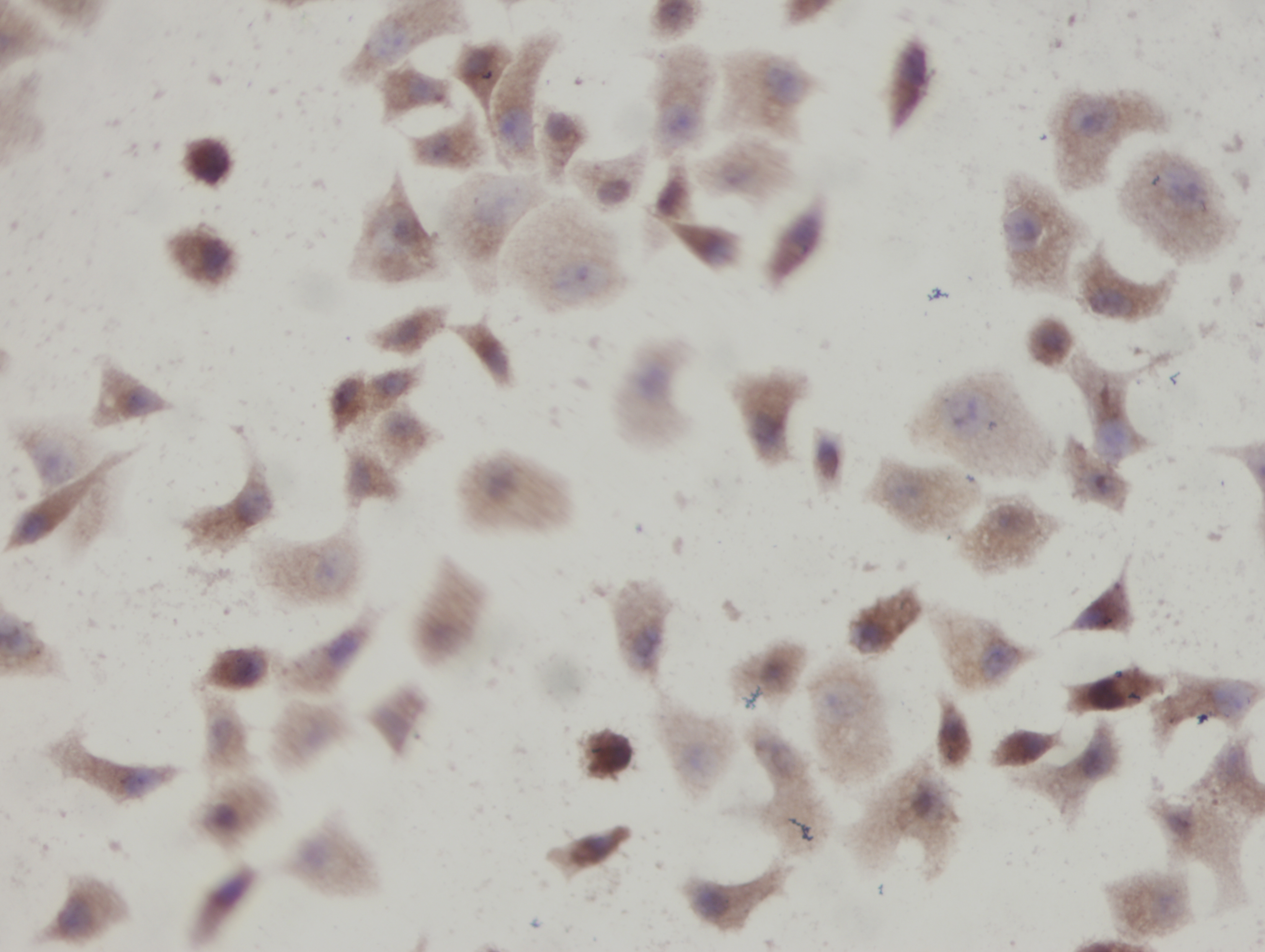

Supplement: Supplementary file 1 — Additional file 1: [file 12885_2023_10543_MOESM1_ESM.zip › Fig6B Hela CEBPB+ CEBPB protein.jpg]

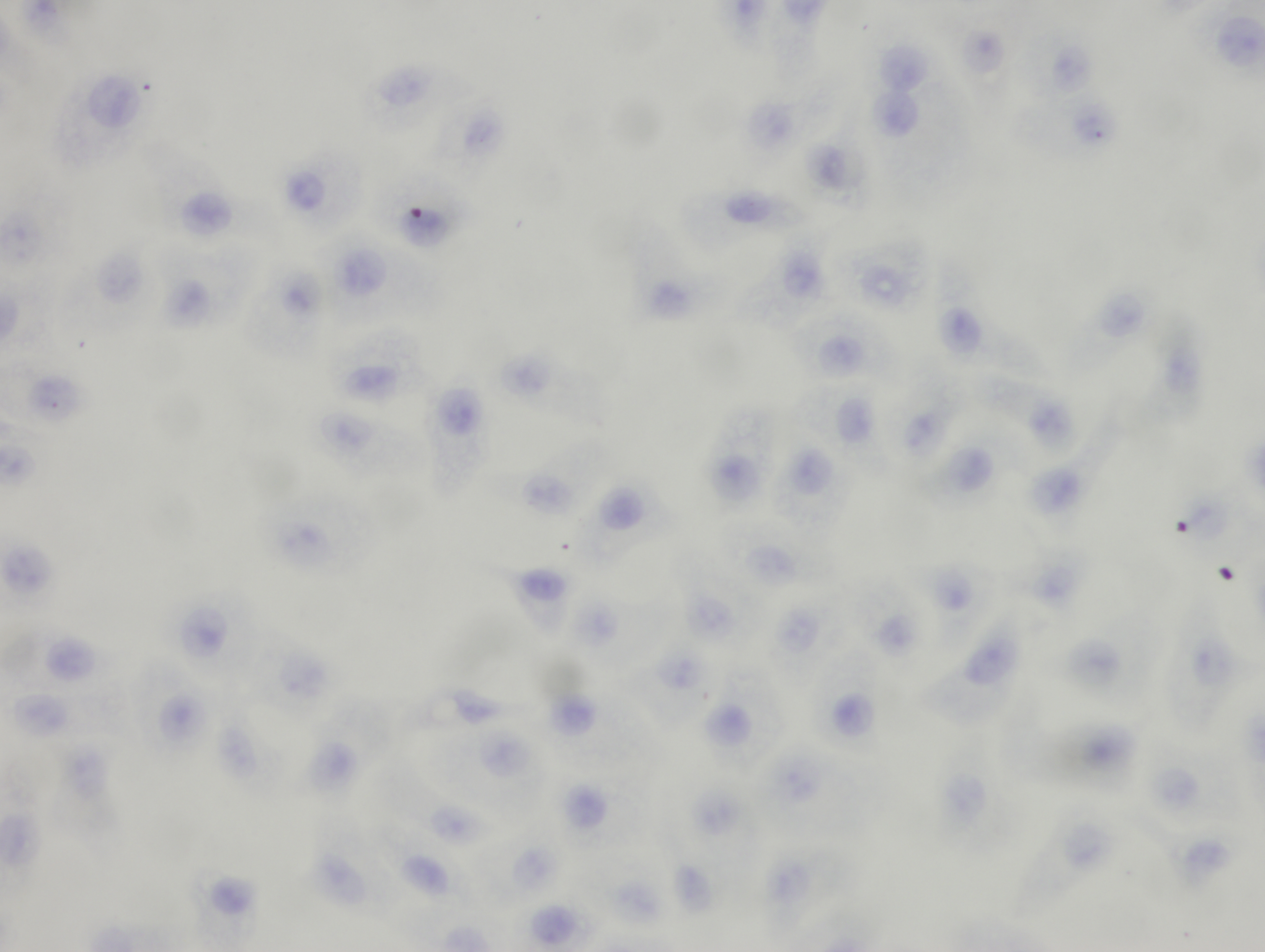

Supplement: Supplementary file 1 — Additional file 1: [file 12885_2023_10543_MOESM1_ESM.zip › Fig6B Hela CEBPB+ Ki67 protein.jpg]

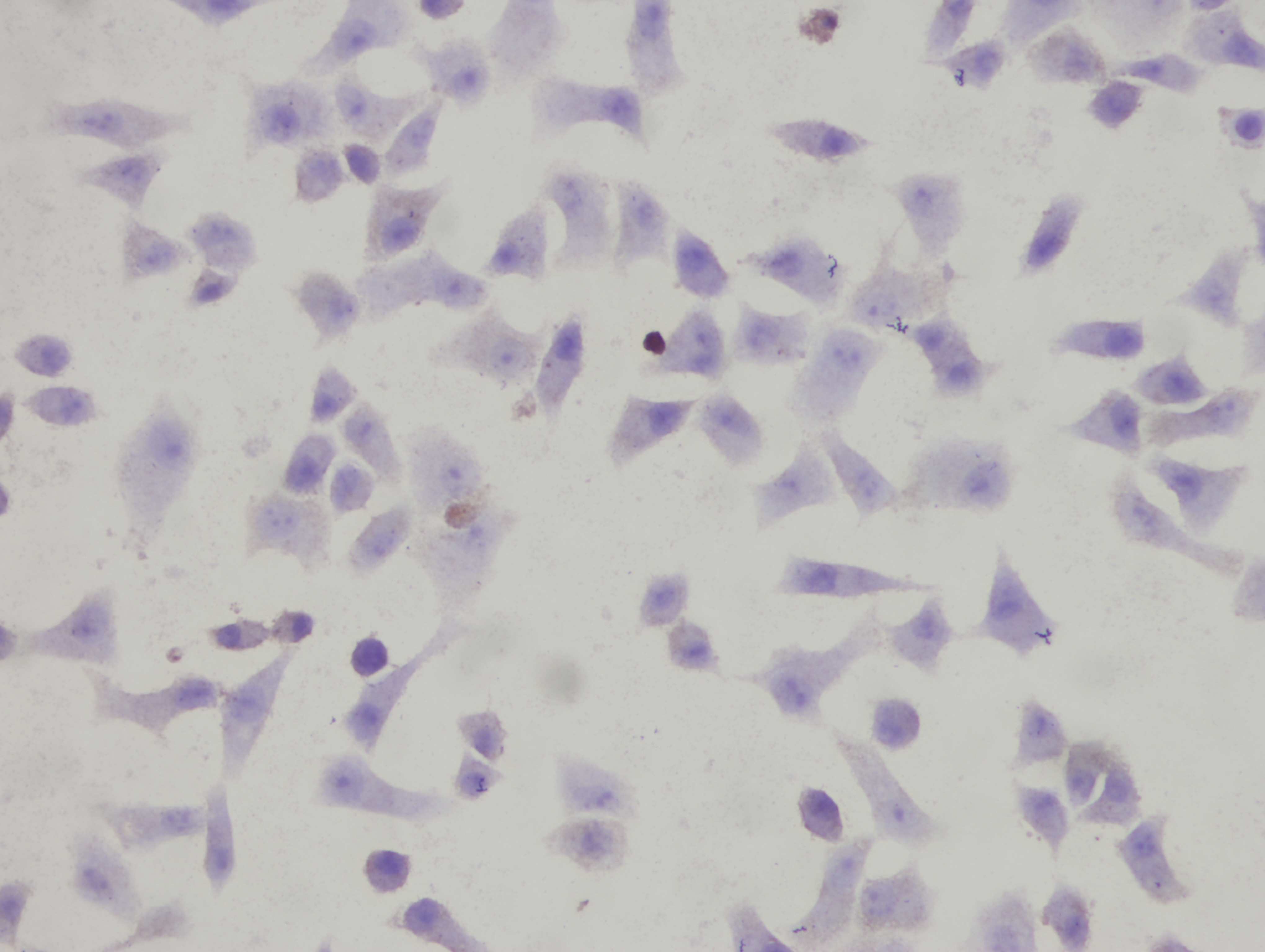

Supplement: Supplementary file 1 — Additional file 1: [file 12885_2023_10543_MOESM1_ESM.zip › Fig6B Hela NC CEBPB protein.jpg]

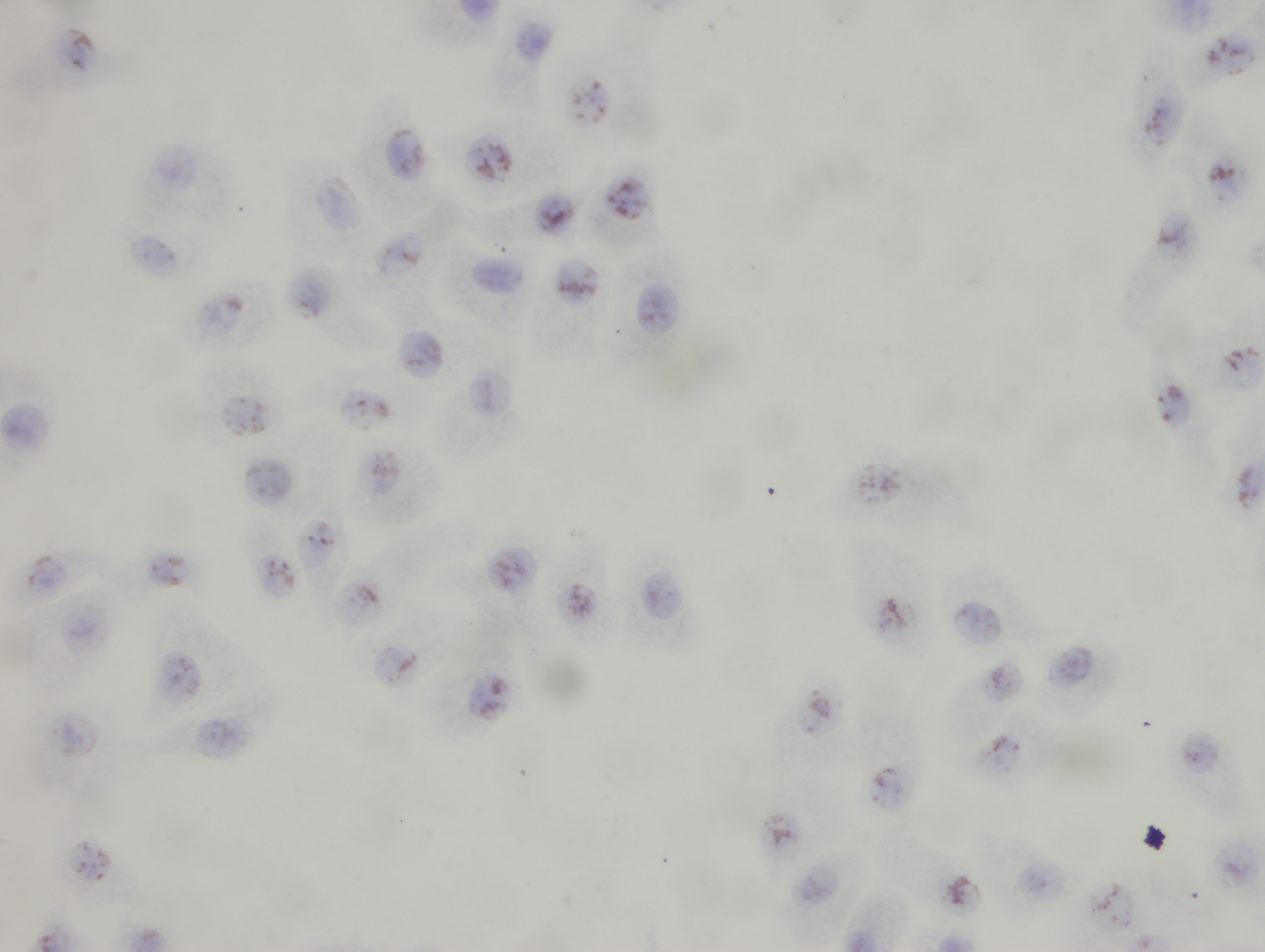

Supplement: Supplementary file 1 — Additional file 1: [file 12885_2023_10543_MOESM1_ESM.zip › Fig6B Hela NC Ki67 protein.jpg]

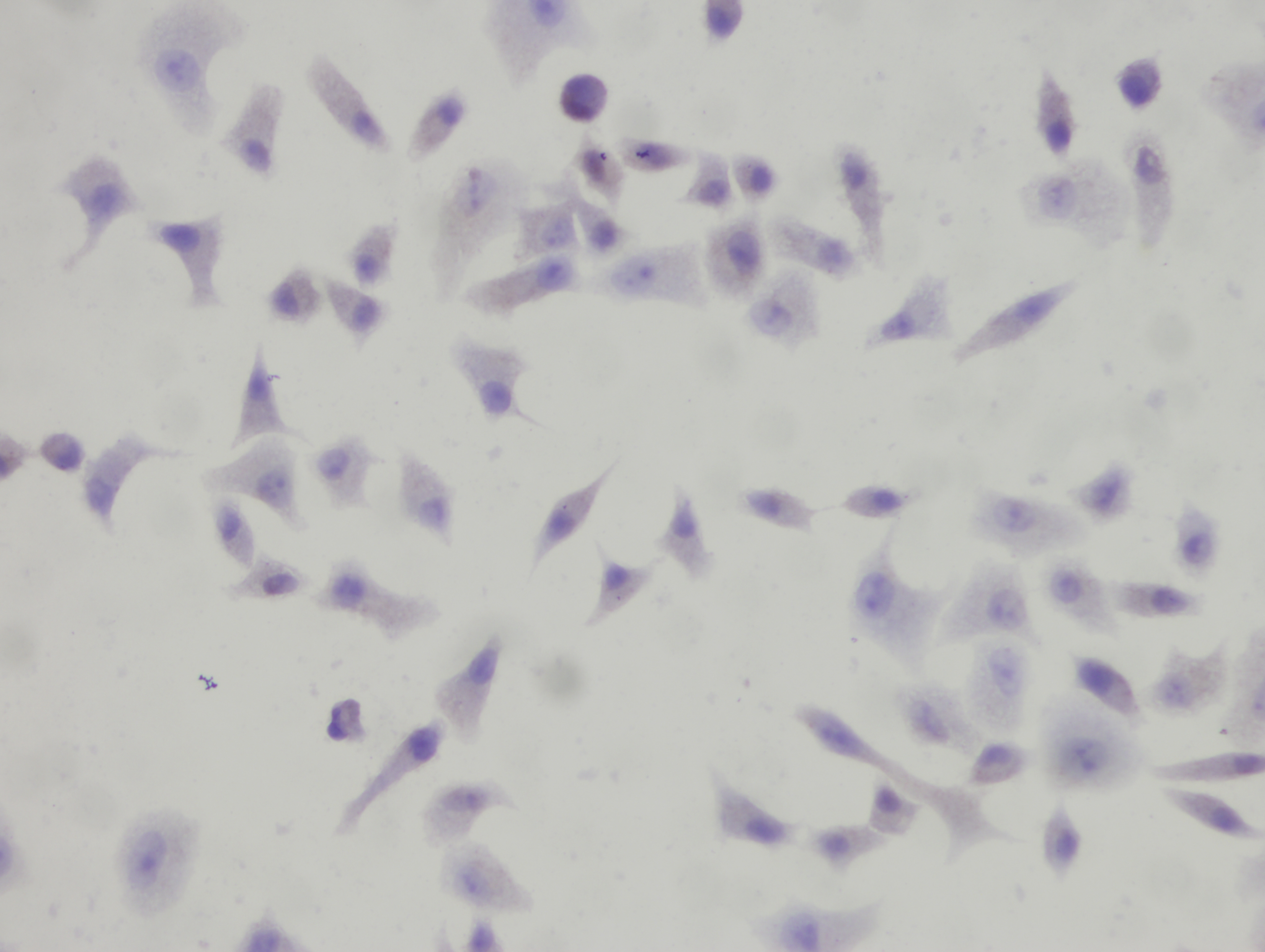

Supplement: Supplementary file 1 — Additional file 1: [file 12885_2023_10543_MOESM1_ESM.zip › Fig6B Hela WT CEBPB protein.jpg]

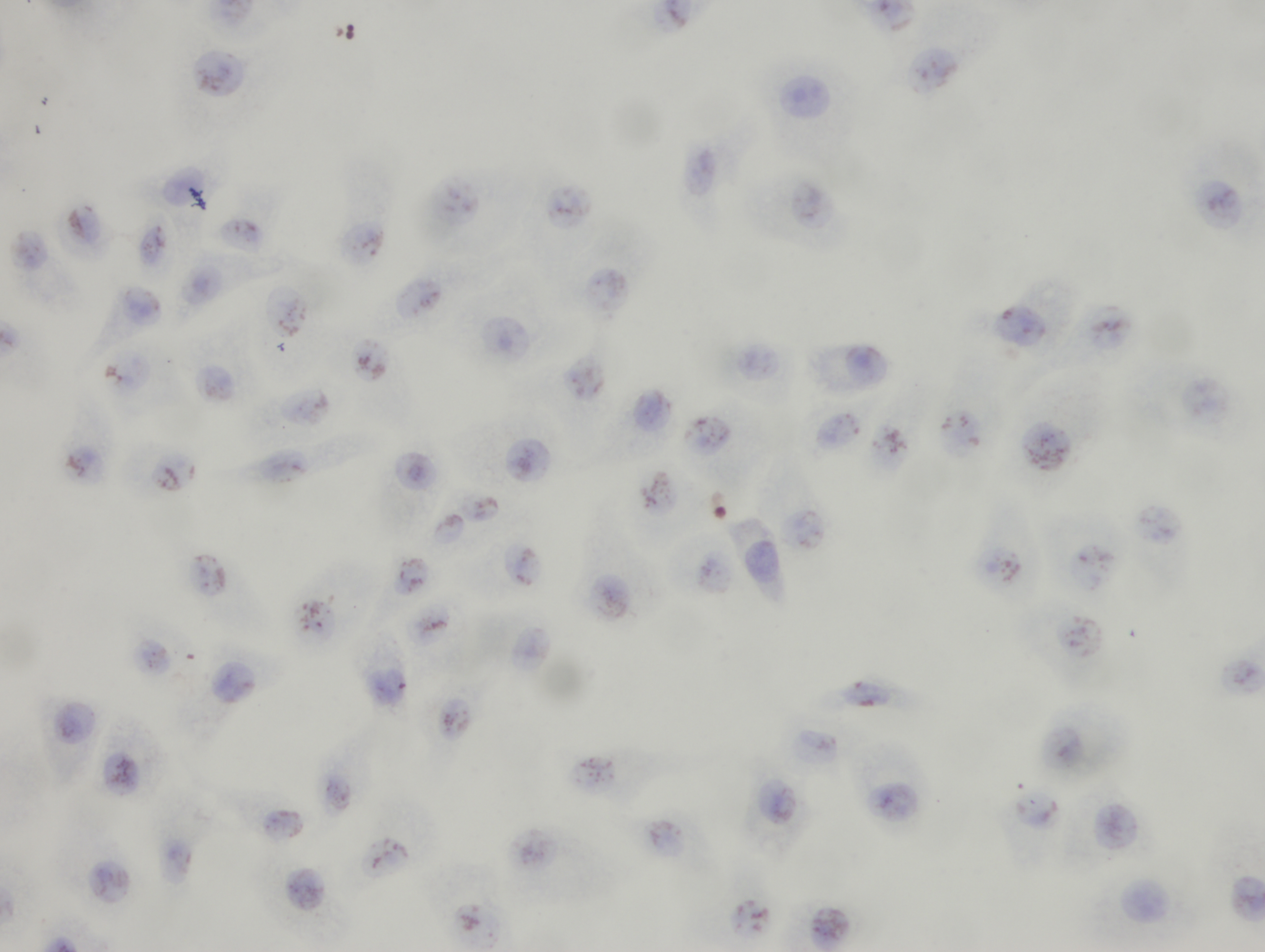

Supplement: Supplementary file 1 — Additional file 1: [file 12885_2023_10543_MOESM1_ESM.zip › Fig6B Hela WT Ki67 protein.jpg]

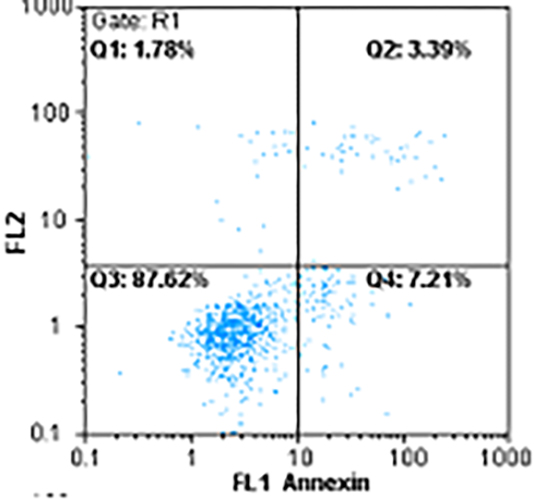

Supplement: Supplementary file 1 — Additional file 1: [file 12885_2023_10543_MOESM1_ESM.zip › Fig6F Hela CEBPB+.jpg]

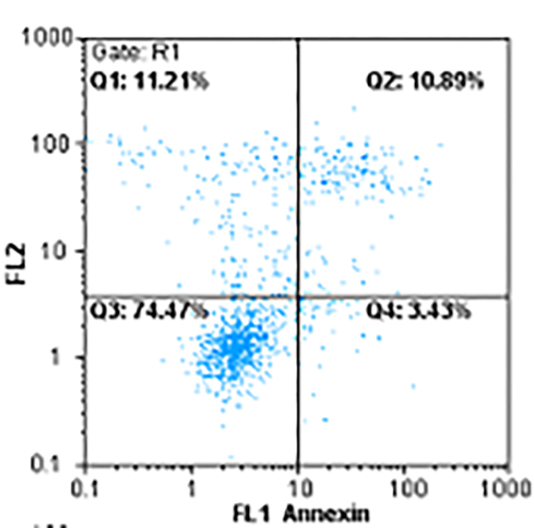

Supplement: Supplementary file 1 — Additional file 1: [file 12885_2023_10543_MOESM1_ESM.zip › Fig6F Hela NC.jpg]
